# Genome-Wide Analysis of Differentially Expressed Genes Relevant to Rhizome Formation in Lotus Root (*Nelumbo nucifera* Gaertn)

**DOI:** 10.1371/journal.pone.0067116

**Published:** 2013-06-26

**Authors:** Libao Cheng, Shuyan Li, Jingjing Yin, Liangjun Li, Xuehao Chen

**Affiliations:** School of Horticulture and Plant Protection of Yangzhou University, Yangzhou Jiangsu, People’s Republic of China; Wuhan University, China

## Abstract

Lotus root is a popular wetland vegetable which produces edible rhizome. At the molecular level, the regulation of rhizome formation is very complex, which has not been sufficiently addressed in research. In this study, to identify differentially expressed genes (DEGs) in lotus root, four libraries (L1 library: stolon stage, L2 library: initial swelling stage, L3 library: middle swelling stage, L4: later swelling stage) were constructed from the rhizome development stages. High-throughput tag-sequencing technique was used which is based on Solexa Genome Analyzer Platform. Approximately 5.0 million tags were sequenced, and 4542104, 4474755, 4777919, and 4750348 clean tags including 151282, 137476, 215872, and 166005 distinct tags were obtained after removal of low quality tags from each library respectively. More than 43% distinct tags were unambiguous tags mapping to the reference genes, and 40% were unambiguous tag-mapped genes. From L1, L2, L3, and L4, total 20471, 18785, 23448, and 21778 genes were annotated, after mapping their functions in existing databases. Profiling of gene expression in L1/L2, L2/L3, and L3/L4 libraries were different among most of the selected 20 DEGs. Most of the DEGs in L1/L2 libraries were relevant to fiber development and stress response, while in L2/L3 and L3/L4 libraries, major of the DEGs were involved in metabolism of energy and storage. All up-regulated transcriptional factors in four libraries and 14 important rhizome formation-related genes in four libraries were also identified. In addition, the expression of 9 genes from identified DEGs was performed by qRT-PCR method. In a summary, this study provides a comprehensive understanding of gene expression during the rhizome formation in lotus root.

## Introduction

Lotus root (*Nelumbo nucifera* Gaertn), which originated from India and China, is an aquatic herb vegetable and a member of the family Nelumbonaceae [1]. It is one of the oldest dicot plants in the world with many features of monocot plants, and has been widely cultivated in China, Japan, and other Southeast Asian counties for multiple purposes [2]. The products of lotus root such as fresh, salted and boiled rhizomes, lotus root starch, drinks, teas, and lotus seeds are very popular in the daily diet because of its richness in nutrients including starch, proteins, vitamins, and mineral substances [3], [4]. China is already exporting the processed products of lotus root to Japan, Korea, Europe, and the United States as a kind of off-season vegetable. In addition, Nodus nelumbinis rhizomatis, germ, stamens, and lotus root stems are also used as important ingredients in the traditional medicine [5], [6], [7].

With the unique characteristics, the rhizome of lotus root is formed underground, and it grows in size after sprouting in the shallow water, such as the pools, water gardens, tanks or tubs of the greenhouse, which indicates that the plant has developed mechanisms of surviving in the submerged environment. Then some floating leaves emerge from the nodes of enlarged and elongated rhizomes. Lotus root produces several rhizomes in a single growing season with average length of 10–20 cm each. Actually, similar with other wetland vegetable (corm, tuber, and bulbs), rhizome is a kind of underground stems, and work as storage organs. These are storage units for food that provide the plants with the energy for growth, blooming, and completing their lifecycle.

Development of storage organ (rhizome) can be classified into four stages: induction (stolon stage), initial swelling, middle swelling, and later swelling stage [8]. Stolon tips grow radically in the induction stage. In the second stages, longitudinal growth of stolon stops and its tip swells [9], [10]. At the middle swelling and later swelling stages, some important carbohydrates are synthesized in the storage organ. For example, accumulation of starch greatly increases in these stages. Environmental factors affect above four stages through triggering signal molecules or gene regulation.

Development of storage organs have been extensively studied, especially in tuber and corm, and great changes have been found in genetic and morphometric processes [12], [13]. Short days (SDP) condition promotes the formation of storage organ, while, long days (LDP) prolongs this process. For example, Masuda et al. (2007) have found that rhizome enlargement was brought about under 8,10 and 12 h photoperiods [14]. The leaf receives photoperiodic signal, and then transports it to the underground stolon tips via the phloem, which promotes the transition of storage organ. CONSTANS, SFT family protein, GIGANTEA, and cycling dof factor are believed to participate in the signal transduction of photoperiodic control, and expression of these genes affects the formation of storage organ [15], [16], [17], [18]. In addition, under SDP condition, StBEL5 represses the gibberellin StGA20 ox1 biosynthesis, which promotes formation of the storage organ [19]. At the same time, the expression of StBEL5 is enhanced by miR172, suggesting that long distance transport of RNA signal also participates in the formation of underground storage organ [20]. In addition, phytochrome B is involved in the response of plants to photoperiodic control, and the formation of storage organ is affected by PHYB in SD. Decreasing the levels of PHYB in transgenic plants promotes the formation of storage organ both in SD and LD, while non-transgenic plants form storage organ only in SD [21]. These results suggest that plants lose the inhibitory effect on formation of storage organ caused by LD when expression of *PHYB* is down-regulated [15]. It is believed that high content of sucrose is required as a necessary condition during the formation of storage organs [15], and sucrose transporters can trigger the formation of storage organ at the early stages [22]. Therefore, sucrose plays an important role at the initial swelling stages of the formation of storage organ [23].

Hormones including: cytokinin, jasmonic acid (JA), gibberellic acid (GA), abscisic acid (ABA), and ethylene are also involved in the initiation and regulation of growth in these storage organs [24], [25], [26]. It has been reported that exogenous application of GA can act as an inhibitor from stolon to induction stage. Transgenic potato plants with GA oxidase gene postpone the storage organ formation. Whereas, decreased expression of this gene results in an earlier formation than non-transgenic plants [27]. Cytokinin and jasmonic acid promote the induction and elongation of storage organ [28]. Bhat et al 29. found that exogenous cytokinin is necessary to induce formation of storage organ in ginger due to improvement in photosynthesis. ABA shows high correlation to tuber formation because ABA-deficient potato plants show retarded tuberization [30]. Exogenous application of auxin on the decapitated peas and potatoes inhibits the formation of axillary buds [31]. Ethylene, produced by almost all plants mediates a variety of developmental processes in plants, such as seed germination, lateral bud stimulation, adventitious rooting, overcoming dormancy, and organ senescence and abscission [26], [32]. Exogenous ethylene is believed as an inducer for the storage organ and root bulking in carrots [33].

Dynamics of rhizome enlargement is relatively poorly known in the lotus root, because detailed investigation of underground rhizome growth is time and labour consuming. Therefore, lotus can be suitable as a model plant to study rhizome growth [14]. Just like the other storage organs, rhizome is also an important edible product, and the developmental processes of this kind of storage organ are regulated by genes [34]. Expressions of these genes affect rhizome formation. Although much work in other species has partially described the above processes, and many storage organ-related genes have been documented [35], but the expression of genes which affect rhizome formation in lotus root have not been studied in detail. Tag sequencing technique has been established as an efficient approach to study gene expression in different environmental conditions [36], [37]. In some vegetable plants, a lot of important genes involved in plant critical metabolisms have been successfully identified by this technique [38], [39], [40], [41]. In this study, DEGs from four developmental stages of lotus root rhizome were sequenced and analyzed with aim to comprehensively understand the processes of rhizome formation and development at molecular level.

## Materials and Methods

### Plant Materials

‘MeiRen Hong’, a wildly cultivated species in China, was planted in the field at the water depth of 20–25 cm in spring with average temperature 30°C/day and 20°C/night during the whole growth season. Three or more stolons were developed and elongated in proper order in each plant. When plants grew up to 4–5 leaves stage (about 90–100 days after plantation), formation of rhizome started at stolon tips. For the analysis of tag-sequencing and gene expression, rhizomes of four developmental stages (stolon, initial swelling, middle swelling and later swelling stage) (Fig. S1) from the plants (three tips from different plants were combined for each stage) were used. To get the materials of different developmental stages, lotus was (rhizome lotus, flower lotus and seed lotus) were cultivated in a field (non-private), located in the South-Eastern China. The permission for sample collection was taken from the Department of Horticulture of YangZhou University, China. No specific permissions were required for the location and the field studies, because the experiments did not involve any endangered or protected species.

### Screening DEGs

Rhizome transcriptome from the above four development stages was analyzed. Stolon tips, rhizomes in the initial swelling, middle swelling and later swelling stages were collected and ground, and the RNA was isolated from the ground samples using RNA extraction mini kit (QIAGEN, Germany). DNaseI was added to eliminate DNA contamination. Sequencing of transcripts in the form of special constructs was completed by Beijing Institute of Genomics (BIG).

To screen the DEGs, transcriptome from these four stages was analyzed with the aspirations to track the major changes in metabolism. RNA was isolated from the materials of these four stages. The DEG libraries of four samples were determined in parallel using Illumina gene expression sample preparation kits. Briefly, the total RNA from four stages was used for mRNA capture with magnetic oligo (dT) beads. The first and second strand cDNA were synthesized, and bead-bound cDNA was subsequently digested with *NlaIII*.

The 3′-cDNA fragments attached to the oligo (dT) beads were ligated to the Illumina GEX NlaIII adapter 1, which contained a recognition site for the endonuclease MmeI for cutting 17 bp downstream of the recognition site (CATG) to produce tags with adapter 1. After removing 3′ fragment via magnetic beads precipitation, an Illumina GEX adapter 2 was introduced at the site of MmeI cleavage. The resulting adapter-ligated cDNA tags were amplified using PCR-primers that were annealed to the adaptor ends for 15 cycles.

The 85 base fragments were purified and recovered by 6% polyacrylamide Trisborate-EDTA gel. The final quality of the tagged sequences was checked by an Agilent 2100 Bioanalyzer. The four tag libraries constructed underwent Illumina proprietary sequencing chip for cluster generation through *in situ* amplification and were deep-sequenced using Illumina Genome Analyzer. For the raw data, we filtered adaptor sequences, low quality tags (tags with unknown nucleotides N), empty reads and tags that were too short or too long, and tags with only one copy to get clean tags. The types of clean tags were represented as the distinct clean tags. Subsequently, we classified the clean tags and distinct clean tags according to their copy number in the library, and showed their percentage in the total clean and distinct tags, and analyzed saturation of the four libraries.

For annotation, all tags were mapped to the reference sequence of NCBI database (http://www.ncbi.nlm.nih.gov/), and no more than 1-bp nucleotide mismatch was allowed. The alignment procedures were conducted essentially by following the protocols described in the online documentation (http://maq. sourceforge.net) and adopting the default parameter values. To monitor mapping events on both strands, both sense and complementary antisense sequences were included in the mapping process. The tags mapped to reference sequences from multiple genes were filtered [38].

### Identification of DEGs

The transcriptome of the lotus root from the above four stages was used as reference for the screening and analysis of the DEGs due to unavailability of the existing data. All expressed genes were monitored, and the gene functions were explored by using database annotations like nr, Swiss-Prot, KEGG, and COG with following criteria: for the gene annotations, blastx alignment (evalue <0.00001) between unigenes and protein databases, such as Swiss-Prot, KEGG, and COG were performed. Best aligning results were used to decide sequence direction and functions of the unigenes.

In case of any conflict in the results from different databases, a priority order of nr, Swiss-Prot, KEGG, and COG was followed when deciding sequence direction of unigenes. When a unigene happened to be unaligned with none of the above databases, ESTscan was used to predict its coding regions as well as to decide its sequence direction. All of the expressed unigenes were classified according to their functions in metabolism processes. For screening the differentially expressed genes, “FDR≤0.001” and the absolute value of “log_2_ Ratio ≥1” were used as a threshold to judge the significance of difference in expression of unigenes. Important genes related to rhizome formation were reposited in NCBI database (TSA: BioProject ID is PRJNA196449; Accession number is GAHV01000000; BioSample is SAMN02028153; Sequence read archive is SRR82669).

### Gene Expression Analysis by qRT-PCR

To carry out the study of gene expression in different species, the cultivation conditions of lotus (rhizome lotus, flower lotus and seed lotus) were kept same as described above. Quantitative RT-PCR analysis was performed to quantify the transcriptional level of nine novel genes with rhizome lotus at stolon stage, initial stage, middle swelling stage and later swelling stage to evaluate the results of tag-sequencing. In addition, expression of 18 important genes at stolon stage, initial stage and middle swelling stage (no obvious later swelling stage in flower and seed lotus) relevant to rhizome formation were also studied in rhizome lotus, flower lotus and seed lotus. Total RNA was extracted from stolon tips, rhizomes of initial swelling, middle swelling and later swelling stage respectively, using RNA extraction mini kit (QIAGEN, Germany). DNaseI was used to digest DNA during the RNA extraction process to eliminate DNA contamination. A total of 1–2 µg of RNA was used in cDNA synthesis according to the manufacturer’s instructions (Promega, USA). The quantitative RT-PCR reaction was performed with the Mx 3000P machine (STRATAGENE, http://www.stratagene.com). The SYBR Green Master Mix was used to identify mRNA level according to the manufacturer’s instructions (Tiangen, China). According to the sequencing results, the primers were designed for the genes that enhanced transcriptional level during rhizome formation which were listed in Table 1. *β-Actin* was used as internal standard and amplified with the primers, forward: 5′-AACCTCCTCCTCATCGTACT-3′, and reverse: 5′-GACAGCATCAG CCATGTTCA-3′. Amplification was performed in a 20 µl reaction mixture, containing 0.16 mM dNTPs, 0.1 µM forward and reversed primers respectively, 1 mM MgCl_2_, 0.4 U Taq polymerase (Tiangen, China), and 1 µl cDNA. The PCR program consisted of 30 cycles: 94°C for 10 min; 94°C for 1 s; 56–60°C for 30 s; 72°C for 60 s, and the final extension at 72°C for 10 min. Triplicate samples were used for quantitative RT-PCR.

**Table 1:** Detailed information about primers used for qRT-PCR variation.

Gene	Forward primer (5′-3′)	Reverse primer (5′-3′)	Tm (°C)	Product (bp)
*CALM*	GAATTGAAGGAAGCGTTTAA	TGGCCAGCATCATTCTCACA	58	200
*SUS*	GCTGATGCTGAATGATCGGA	CATCAGGTATTAAACGGGTC	56	357
*GA20ox*	TGTCTCCTAATGGATTCAAC	CTCGTCACCGTTGAGGAATC	52	225
*ERF*	GGAATGGAAGGGTCCGATGT	CATGAGATACACTTTCTAACT	52	354
*bZIP*	CGCCACCTTGAGTCAGCTGT	CAGTGTCTGGGAGTCGGTCA	55	276
*WRKY*	CTCTTTATCCAGCATCTTTGA	GGTTCTCTCTGCCGTTTCTG	54	348
*SAUR*	ATGGCTCTCAGGAAATCAAA	ATAGTGAGACCCATGTCGTG	53	266
*CBF*	AGAGGAGTCAGGGAGAGGA	GCAACCTTCTCTTCGCTCTC	57	329
*GBSS*	TGGGGAACACTTCAATATCT	CTCCCGAAGAGGAAGACCA	52	307
*CONSTANS*	GGCATTCCTTGCGATTTCTG	TCGGAGCTGCAATTGTCG	55	182
*GIGANTEA*	AGAACTCAAGCCACCTACTA	GGTAGCAGTTTCATAGCGAG	53	153
*MADS*	CTGAGGTCGCTGTGATCGT	GCCTTAAGTTTGGTGTGTTC	52	178
*Dof*	CAATCTTTCCCAACCTCG	ACGAAGACAAGGACGATGA	53	168
*HD*	CGCCGTCTGCTCCAACAA	CGATGAAAGTTCCTGTGGCA	60	200
*Lipoxygenase*	CCCTTTGCCTCAAGTTATCC	GCAGCCCATTCAGGTTATTC	55	206
*GP*	AGGTTTGGGCACTACTATGG	TTAGGGTATCATCATGCGTG	54	173
*PHYB*	GTGGAATGCAGCAATGGA	ATCCACATATTTTCCTTTCC	51	211
*APETALA*	GTCAGTCGGAACCTCTTGC	CCGCCCAAATAAACCTGC	54	175

All primers used for qPCR were designed according to CDS obtained in this study in lotus root.

## Results

### 2.1 Transcriptome of Rhizome

Four stages of rhizome development: stolon stage, initial swelling, middle swelling and later swelling stages were selected to construct libraries using the Illumina sequencing platform to investigate the genes involved in rhizome formation. A total of 49053 genes which included 39497 (accounted for 80.52%) CATG site genes used as reference genes were obtained after the transcriptome of above four stages were preprocessed.

About 5 million raw tags in each library were obtained with 327019, 312178, 363183, and 385558 raw distinct tags respectively. To get clean tags, all the raw tags were filtered with reference sequences, and 4542104, 4474755, 4777919, and 4750348 clean tags including 151282, 137476, 215872, and 166005 distinct tags in L1, L2, L3, and L4 libraries were obtained respectively. All data of tags for each library is given in Table 2. To testify whether or not the sequenced tags were sufficient to cover the whole transcriptome, the analysis of sequencing saturation was also performed in the four libraries. The number of detected genes increased until the sequencing tags reached 3 million or more, which indicated that the identified expressed tags were enough to reflect the whole transcriptional information in genome of lotus root (Fig. S2).

**Table 2:** Categorization and abundance of tags.

		L1	L2	L3	L4
Raw Tag	Total number	4722270	4654357	4931007	4975127
	Distinct Tag	327019	312178	363183	385558
Clean Tag	Total number	4542104	4474755	4777919	4750348
	Distinct Tag number	151282	137476	215872	166005
All Tag Mappingto Gene	Total number	3593944	3550079	3833530	4043129
	Total percentage of clean tag	79.13%	79.34%	80.23%	85.11%
	Distinct Tag number	91450	81334	115876	108718
	Distinct Tag percentage of clean tag	60.45%	59.16%	53.68%	65.49%
Unambiguous TagMapping to Gene	Total number	2918451	2919531	3315975	3300501
	Total percentage of clean tag	64.25%	65.24%	69.40%	69.48%
	Distinct Tag number	73493	64898	93692	87374
	Distinct Tag percentage of clean tag	48.58%	47.21%	43.40%	52.63%
All Tag-mappedGenes	Total number	26261	24551	29314	27666
	Percentage of reference genes	53.54%	50.05%	59.76%	56.40%
Unambiguous Tag-mapped Genes	Total number	20471	18785	23448	21778
	Percentage of reference genes	41.73%	38.30%	47.80%	44.40%
Unknown Tag	Total number	948160	924676	944389	707219
	Total percentage of clean tag	20.87%	20.66%	19.77%	14.89%
	Distinct Tag number	59832	56142	99996	57287
	Distinct Tag percentage of clean tag	39.55%	40.84%	46.32%	34.51%

About 5.0 million tags were sequenced, and clean tags and distinct tags were obtained after removal of low quality tags from each library. Some distinct tags were unambiguous tags after mapping to the reference genes, and some were unambiguous tag-mapped genes.

Functions of gene were annotated by comparison against existing NCBI database.

**Figure 1:**
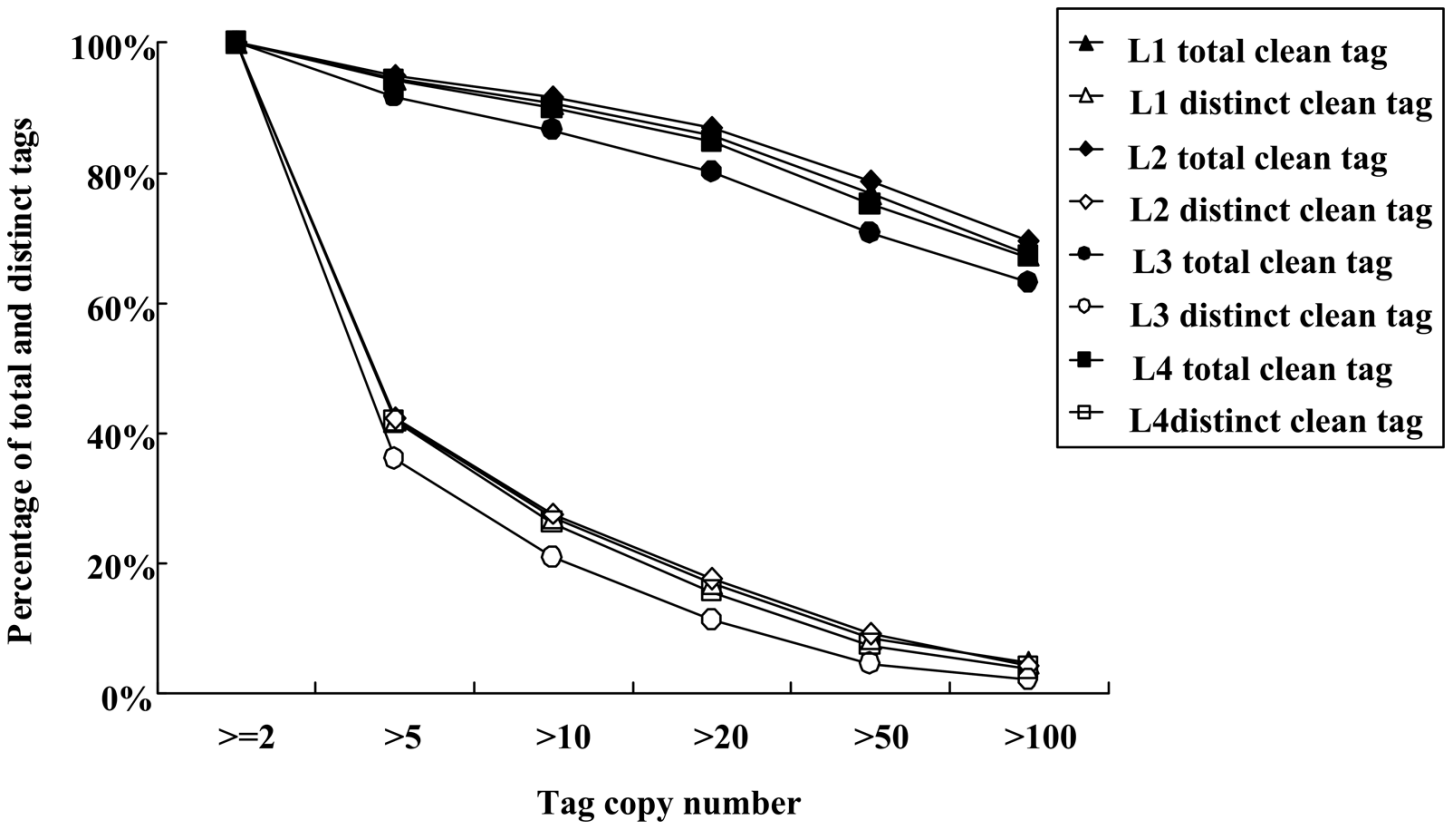
Distribution of total clean tags and distinct clean tags count from the four libraries.

Among which, more than 41% of all distinct sequences in four libraries showed an above cut-off BLAST result, and about 59% could not match with the known genes. These identified genes were classified into 26 catalogues according to their functions. One catalogue containing the largest number of genes predicted functions, and the smallest one was relevant to nuclear structure (Fig.S3). The copy distribution of total and distinct clean tags in four libraries showed the same tendency with about 4% of distinct clean tags higher than 100 copies and more than 11% tags being in 20–50 counts. The number of distinct clean tags between 2–5 copies (approximately 40%) was high as compared with that of others (Fig.1).

### 2.2 DEGs in Four Libraries

#### Gene expression profiles during rhizome formation

**Figure 2:**
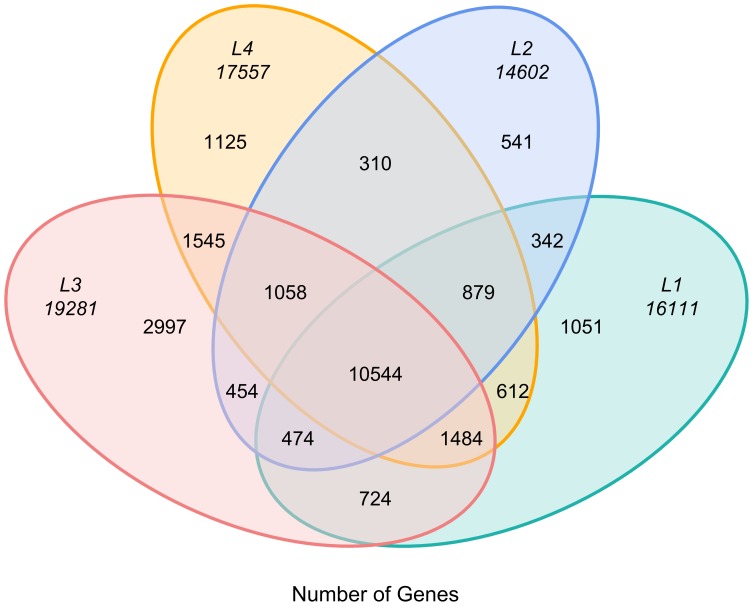
Analysis of tag mapped genes among four libraries.

Gene expression profiles during rhizome development in four libraries were examined. Total 16111, 14602, 19281, and 17557 transcripts were identified from L1, L2, L3, and L4 libraries respectively. We found that 10544 genes were expressed in all four libraries, and 1051, 541, 2997, and 1125 were especially expressed in L1, L2, L3, and L4 libraries respectively (Fig. 2).

Differentially expressed genes were identified from these four different developmental stages during rhizome formation to uncover the changes in metabolism at molecular level. The abundance of transcripts in four libraries was counted by the number of genes per million clean tags (TPM). FDR (false discovery rates) <0.001 and the absolute value of |log2 Ratio|≥1 were used as a threshold to judge whether gene expression was significant. A total of 1714 genes were found to change their transcriptional level in L1/L2 libraries. From which, 759 were up-regulated (Table S1) and 955 were down-regulated. In L2/L3 libraries, 4013 genes, including 2157 up-regulated genes and 1856 down-regulated genes were found. In L3/L4 libraries, 2344 genes were observed to change their transcription including 1429 up-regulated genes and 915 down-regulated genes (Table 3). According to the gene expression profile in L1/L2, L2/L3, and L3/L4 libraries, a large number of genes showed altered mRNA transcriptional levels in L2/L3 libraries. However, compared with other libraries, number of transcripts in L1/L2 libraries showed a little change.

**Table 3:** DEGs across all libraries.

	L1:L2	L1:L3	L2:L3	L3:L4	L1:L4	L2:L4
Total	1714	4339	4013	2344	2115	1667
Up-regulated	759	2052	2157	1429	1069	1059
Down-regulated	955	2287	1856	915	1046	608

All the genes mapped to the reference sequence and genome sequences were examined for their expression differences across different libraries. Numbers of differentially expressed genes represent transcripts, using threshold values FDR≤0.001 and |log2 Ratio|≥1 for controlling false discovery rates. L1, L2, L3, and L4 represent the samples which were collected at stolon stage, initial swelling stage, middle swelling stage, and later swelling stage of rhizome, respectively.

#### Most DEGs in each library

The variation in expression was observed in 1714, 4013, and 2344 genes in L1/L2, L2/L3, and L3/L4 libraries, respectively. Therefore, 20 DEGs with higher levels of changes were selected respectively, to monitor changes in metabolism during rhizome formation. We found that the most DEGs in L1/L2 libraries were Pectinesterase, a ubiquitous cell-wall-associated enzyme that facilitates plant cell wall modification and subsequent breakdown. DEGs in L1/L2 libraries could be mainly classified into 3 catalogues which belonged to hormone response (SAUR family protein and Ethylene responsive transcription factor), fiber synthesis (Glycerophosphoryl diester phosphodiesterase, Receptor protein kinase-like protein, G-type lectin S-receptor-like serine, Fiber expressed protein, Calcium-dependent protein kinase, Sucrose synthase) and stress response proteins (Ethylene responsive transcription factor, Calcium-dependent protein kinase, Peroxidase, SAUR family protein). In addition, an extensin and vacuolar-processing enzyme was also found to be involved in the process from stolon stage to initial swelling stage (Table 4). DEGs in L2/L3 libraries could be mainly classified into four catalogues. One catalogue was associated with energy metabolism (NADH dehydrogenase, Malate dehydrogenase [NADP], ATP synthase subunit beta). The second was relevant to synthesis and transport of matter (Starch-branching enzyme I, Sugar transporter, 3-ketoacyl-CoA synthase 4, 2-methyl-6-phytylbenzoquinone methyltranferase, Vacuolar H+-pyrophosphatase),DEGs in third catalogue were involved in hormone metabolism and response (Auxin response factor 2, Ethylene-responsive transcription factor ERF114, ACC oxidase, Xanthine dehydrogenase). The fourth catalogue of DEGs covered was stress response genes (Transcription factor bHLH80, Vacuolar H+-pyrophosphatase). In L3/L4 libraries, DEGs were classified into three major catalogues, such as synthesis of matter (Basic 7S globulin, UDP-glycosyltransferase 89B2, Sucrose synthase, UDP-glycosyltransferase, Glutathione S-transferase, Fiber expressed protein, Patatin group), hormone synthesis and response (Gibberellin-regulated protein 6, Gibberellin 20 oxidase, Ethylene-responsive transcription factor ERF025, ERF3 transcription factor) and swelling related proteins (Alpha-expansin 3, Extensin). In addition, we also found ATPase subunit enhanced transcriptional level in later swelling stage. A lot of genes were down regulated in L1/L2, L2/L3, and L3/L4 libraries, most of which in L1/L2 libraries were involved into synthesis of matter, and others in L2/L3 and L3/L4 libraries were relevant to cell growth and differentiation, translation, RNA process, and modification.

**Table 4:** 20 most differentially expressed annotated genes in L1/L2, L2/L3, and L3/L4 libraries based on expressed tag frequency.

Gene ID	Relative abundant (TMP ratio)	Function annotation	Fragment (bp)
**L1/L2** libraries			
L21966	15.747	Pectinesterase-2 precursor	1068
L6372	12.111	Non-specific lipid-transfer protein	558
L4842	11.182	Primary amine oxidase-like	355
L19738	10.569	Inositol oxygenase	924
L5740	9.611	MLP-like protein	28 456
L5559	9.525	Glycerophosphoryl diester phosphodiesterase	489
L9661	9.26	Extensin (class I)	198
L4093	9.23	Formamidase	291
L11656	9.12	Vacuolar-processing enzymeprecursor	1464
L14179	8.94	Receptor protein kinase-like protein	402
L24462	8.93	Chaperone protein dnaJ 8	234
L19479	8.81	CytochromeP45082C4	1479
L22249	8.8	Peroxidase 64-like isoform 2	933
L9369	8.73	Rop guanine nucleotide exchange factor 2	219
L13340	8.65	G-type lectinS-receptor-likeserine	1484
L15631	8.41	Fiber expressed protein	345
L16154	8.33	Ethylene responsive transcription factor	927
L7272	8.29	SAUR family protein	234
L3989	8.28	Calcium-dependent protein kinase	783
L2349	8.20	Sucrose synthase	519
L2/L3 libraries			
L9592	13.40	Cytochrome P450 86A1	1530
L3363	12.66	Phytylbenzoquinone methyltranferase	810
L19686	11.81	3-ketoacyl-CoA synthase4	1499
L24558	10.83	CASP-like protein RCOM_1174750-like	480
L14401	10.58	GDSL esterase/lipase At2g23540-like	743
L13523	9.98	Transcription factor bHLH80	450
L9063	9.97	Auxin response factor 2	711
L11556	9.67	E3 ubiquitin-protein ligase UPL4-like	699
L3219	9.43	Starch-branching enzymeI	2205
L6212	9.19	NADH dehydrogenase	291
L4985	9.14	Sugar transporter	1470
L2123	8.98	Calcineurin subunit B isoform 1	231
L2369	8.97	Vacuolar H+-pyrophosphatase	300
L21145	8.96	ABC transporter family protein	420
L4307	8.95	Ethylene-responsive transcription factor ERF114;	804
L18002	8.90	Xanthine dehydrogenase	486
L26121	8.67	Malate dehydrogenase [NADP]	254
L6411	8.44	ATP synthase subunit beta	1461
L7701	8.40	Calcium-dependent protein kinase 30	1005
L378	8.30	1-aminocyclopropane-1-carboxylate oxidase	822
L3/L4 libraries			
L22026	13.72	Basic 7S globulin	962
L18835	12.626	Omega-hydroxypalmitate O-feruloyl Transferase	1361
L134	11.97	Alpha-expansin 3	750
L4954	11.91	Extensin	513
L6449	11.01	Gibberellin-regulated protein 6	315
L3691	10.98	Gibberellin 20 oxidase	289
L3566	10.56	Ethylene-responsive transcription factor ERF025	393
L19429	10.48	ATPase subunit	285
L24010	10.41	F-box family protein	1266
L4407	10.40	Heat shock protein70	1188
L15631	10.39	Fiber expressed protein	345
L7453	10.12	Glutathione S-transferase	411
L8862	10.09	UDP-glycosyltransferase89B2	1107
L2616	9.62	Sucrose synthase	1131
L3393	9.56	Cell wall-associated hydrolase	495
L309	9.39	ERF3 transcription factor	498
L15819	9.19	UDP-glycosyltransferase 88A	1407
L23091	9.15	EF-hand calcium-binding protein	516
L8032	8.97	WD repeat-containing protein 18	627
L10302	8.75	Patatin group A-3	1014
**L1/L2 libraries**			
L16154	8.29	Ethylene-responsive transcription factorERF017	348
L1375	8.18	CBF/DREB3 transcription factor	609
L17409	8.04	Transcription factor bHLH35	726
L22245	7.81	AP2 domain class transcription factor	923
L5201	7.80	WRKY transcription factor 6-like	666
L161	7.31	KNAT3-like transcription factor	747
L7327	6.81	bZIP transcription factor 60	495
L3810	6.47	GRAS family transcription factor	308
L959	6.46	Transcription factor hy5	435
L24021	6.06	MYB transcription factor	594
L5723	6.05	MADS-box transcription factor	744
L16316	5.52	Transcription factor TCP9	1215
L4285	5.49	Heat stress transcription factor A-4b	960
L10423	5.48	NAC domain-containing protein	471
**L2/L3 libraries**			
L13523	9.98	Transcription factor bHLH80	450
L4307	8.95	Ethylene-responsive transcription factor ERF114	804
L7947	8.19	Homeobox protein BEL1 homolog	336
L3540	8.100	Trihelix transcription factor GT-1	972
L9587	7.97	NAC domain protein NAC1	710
L1325	7.71	Transcription factor ICE1	477
L7126	6.70	Transcription factor MYB59-like	345
L3348	6.98	Transcription factor SPATULA	801
L21444	5.55	NF-X1-type zinc finger protein NFXL1-like	2277
L6302	5.54	KANADI like transcription factor	834
L545	5.52	WRKY transcription factor 44	1125
L4641	5.2	Scarecrow-like transcription factor PAT1-like	1644
L20454	4.71	BZIP domain class transcription factor 60	495
L1140	4.70	Calmodulin-binding transcription factor SR3L	345
L25749	3.92	GATA domain class transcription factor	279
L12403	3.39	HSF transcription factor	384
L6635	3.36	HD domain class transcription factor	258
L5314	2.35	Transcription factor KAN2	447
L25842	1.34	Zinc finger protein CONSTANS-LIKE	894
**L3/L4 libraries**			
L3566	10.56	Ethylene-responsive transcription factor ERF025	393
L5181	10.40	HSP transcription factor	384
L309	9.39	ERF3 transcription factor	498
L18531	8.55	Transcription factor HEC1	699
L6220	7.19	WRKY transcription factor 70	951
L5632	6.98	Transcription factor AS1	1071

In this study, some important transcription factors involved in rhizome formation were also identified. A total of 14, 19, and 20 transcription factors were found in L1/L2, L2/L3, and L3/L4 libraries, respectively (Table 5). In L1/L2 libraries, some of transcription factors were ethylene-responsive which include: ERF017, CBF/DREB3, bHLH35, TCP9, and WRKY transcription factors, and AP2 domain class transcription factor, KNAT3-like transcription factor, bZIP transcription factor 60, GRAS family transcription factor, transcription factor hy5, MYB transcription factor, MADS-box transcription factor, heat stress transcription factor A-4b, and NAC domain-containing protein. In L2/L3 libraries, expression levels of many important regulators (e.g. transcription factor bHLH80, homeobox protein BEL1 homolog, ethylene-responsive transcription factor ERF114, trihelix transcription factor GT-1, NAC domain protein, transcription factor ICE1, transcription factor MYB59-like, transcription factor SPATULA, NF-X1-type zinc finger protein NFXL1-like, KANADI like transcription factor, WRKY transcription factor 44, scarecrow-like transcription factor PAT1-like, BZIP domain class transcription factor, calmodulin-binding transcription factor SR3L, GATA domain class transcription factor, HSF domain class transcription factor, HD domain class transcription factor, transcription factor KAN2, and zinc finger protein CONSTANS-LIKE) were enhanced. In L3/L4, most of the genes were found to be involved in matter metabolism and stress response.

**Table 5:** Expression abundance of up-regulated transcriptional factors during rhizome formation.

L3982	6.97	GATA transcription factor	621
L18475	6.71	Dof zinc finger protein	630
L14316	6.70	NF-X1-type zinc finger protein NFXL1	2277
L1375	6.38	CRT/DRE binding factor 1	681
L2135	5.98	NAC transcription factor	471
L19946	5.96	BZIP transcription factor 107	81
L21009	5.39	ZF-HD homeobox protein	705
L5026	5.38	Transcription factor PIF3	2184
L5723	5.37	MADS-box transcription factor	744
L12725	4.32	NAC domain protein	471
L6472	3.11	MYB transcription factor MYB2	411
L22833	2.51	BEL1-like home domain protein 1	1244
L2868	2.44	Scarecrow-like protein 4	1818
L13679	1.37	Transcription initiation factor TFIID	587

#### Genes related to rhizome formation

To test whether the transcription patterns in this study had coverage of the well-defined genes, the data sets of this experiment were compared to previous reports. We found 14 identified genes relevant to rhizome formation. The expression of these genes and their biological functions are listed in Table 6. Among which, 8 genes including zinc finger CONSTANS-like protein, GIGANTEA (clock-regulated protein), BEL1-like HD transcription factor, sucrose synthase, ca^2+^/Calmodulin-like protein, lipoxygenase, glucose pyrophosphorylase, granule-bound starch synthase, phytochrome B enhanced their transcriptional levels, and 3 genes (SFT family protein, cycling dof Factor) did not show any significant change in its expression in all the four libraries. Three genes [MADS-box transcription factor, GA 20-oxidase (aside from L3/L4 stages), APETALA1)] directly decreased their expression during the rhizome development.

**Table 6:** Expressed abundance of some rhizome formation-related genes identified previously.

Gene ID	TPM-L1		TPM-L2	TPM-L3	TPM-L4	Function annotation		References
L20523		5.28	7.82	20.93	10.95		Zinc finger CONSTANS-like protein	[17]
L21782		0.88	2.01	4.6	1.89		GIGANTEA (clock-regulated protein)	[20]
L6549		27.08	21.05	18.42	20.0		MADS-box transcription factor	[84]
L9854		1.1	1.45	2.26	1.26		SFT family protein	[124]
L22473		7.93	6.7	6.28	6.74		Cycling Dof Factor	[125]
L29236		1.32	2.46	7.53	3.37		BEL1-like HD transcription factor	[126]
L2349		0.01	0.45	3.35	0.01		Sucrose synthase	[90]
L16799		0.01	1.79	1.26	1.26		Calmodulin-like protein	[93]
L18271		0.44	2.01	9	7.37		Lipoxygenase	[127]
L5098		76.18	73.63	135.62	154.09		Glucose pyrophosphorylase	[128]
L19795		0.01	0.45	11.93	1.68		Granule-Bound Starch Synthase	[129]
L16933		0.01	0.89	3.98	2.74		Phytochrome B	[130]
L18298		0.44	0.22	0.12	1.45		GA 20-oxidase	[27]
L4398		0.44	0.03	0.09	0.04		APETALA	[131]

TMP, transcripts per million clean tags.

### 2.3 Expression Analysis of Nine Genes through qRT-PCR

**Figure 3:**
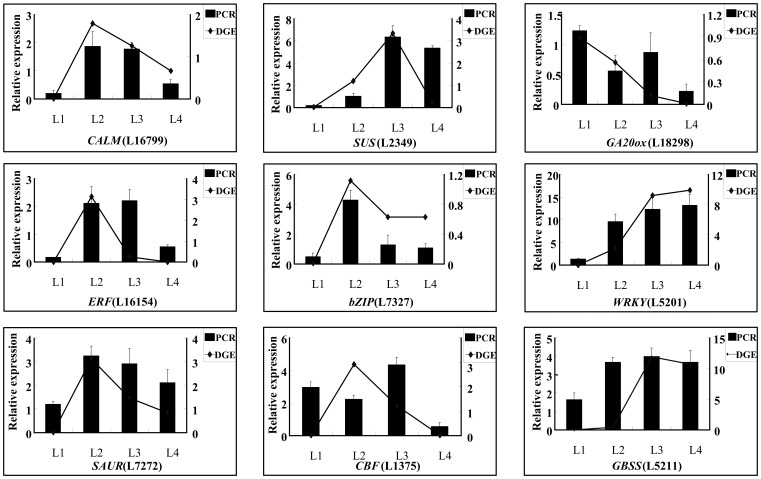
Validation of tag-mapped genes from three stages of lotus root with quantitative RT–PCR.

Nine genes including *CALM*, *SUS*, *GA20ox*, *ERF*, *bZIP*, *WRKY*, *SAUR*, *CBF*, and *GBSS* involved in rhizome formation were studied by quantitative RT-PCR method. From the data of results, expression profiling of seven genes in four developmental stages i.e. stolon, initial swelling, middle swelling, and later swelling stage by qRT-PCR showed similar tendency as found in Tag-sequencing, which indicates a correspondence of the results from qRT-PCR analysis with the Tag sequencing analysis. Only two genes (*CBF* and *GBSS*) were observed with some difference in L2/L3 libraries in transcriptional level between Tag-sequencing analysis and qRT-PCR data (Fig. 3). In addition, detailed study for the expression of 18 genes in rhizome lotus, flower lotus and seed lotus was also carried out with qRT-PCR method. The results showed that many genes including *bZIPs*, *WRKY*, *GBSS*, *CONSTANS*, GIGANTEA, *Dof, HD*, *PHYB*, *Lipoxygenase* and *GP* showed different expression profiles in three phenotypes of lotus. Among which, expression of *CONSTANS*, GIGANTEA, *Dof, HD*, *PHYB*, which involved in photoperiodic signals was higher in flower lotus and seed lotus than that of rhizome. Whereas, *GBSS* and *Lipoxygenase bZIPs*, *WRKY* and *GP* showed higher transcriptional level in rhizome as compared with that of flower lotus and seed lotus (Fig. S4).

## Discussion

### Tags Identified in Four Stages of Lotus Rhizome

Recently, a lot of regulatory mechanisms have been identified by high throughout tag-sequencing technique [38], [39], [41], [42]. Although a large number of genes have not been annotated, tag-mapped genes have been believed to fully cover the whole plant genome [39]. It is an evidence that enlargement of lotus rhizome (*Nelumbo nucifera*) is strictly regulated by environmental factors, especially photoperiodic response can affect rhizome morphogenesis [14]. Therefore, in this experiment, lotus root was cultivated in an open field with normal environmental factors. In addition, rhizomes at four obvious differential developmental stages including stolon stage, initial stage, middle swelling stage, and later swelling stage were used due to few reports on the rhizome transition. Genes with differential expression during rhizome formation were identified using this technique. Approximately 5.0 million tags were identified per library, 4542104, 4474755, 4777919, and 4750348 clean tags were obtained. Only 20471, 18785, 23448, and 21778 genes from L1, L2, L3, and L4 libraries respectively, could be annotated, due to unavailability of complete genome of lotus root (Table 2). We found that many genes significantly changed their expression during rhizome development. The higher number of DEGs in L2/L3 libraries (Table 3) suggests that regulation from the initial swelling stage to middle swelling stage is more complex as compared with that of other developmental stages.

### Identification of DEGs in Four Libraries

DEGs in four libraries were involved in the transport signal transduction, small molecular biosynthesis, transcription, cell cycle, response to the stimuli, organelle organization, anatomical structure development, cell differentiation, translation, organ development, cellular macromolecular metabolic processes, energy, and cellular component organization (Fig. S3). In L1/L2 libraries, 20 most DEGs are involved in hormone response, fiber synthesis, and response to stresses. In L2/L3 libraries, however, the expression profiles of major DEGs were relevant to the energy metabolism, synthesis and transport of storage, hormone metabolism and response, stress response and energy metabolism. In L3/L4 libraries, most DEGs were involved in storage synthesis, hormone synthesis and response. All genes which showed enhanced expression in these four libraries were listed in Table S1 (Table S1).

### Adaptation to Submergence Stress

Adaptation to submergence is very important for the survival of lotus root. In this study, we found that several genes which included (ADH (L15773), MYB (L24021), NADH dehydrogenase (L6212), SOD (L3610), Ethylene-responsive transcription factor (L16154), Calcium-dependent protein kinase (L3989), ATPase subunit (L6411), heat shock protein (L4407) related to submergence enhanced their expression during rhizome formation (Table 1 and Table S1). Some species such as soybean, barley, tomato, and corn are also known to be sensitive to flooding [43], [44], [45], [46], and their productivity is seriously affected due to exposure to anaerobic stress. However, most aquatic plants (including lotus root) exposed to submergence show high resistance [45], [47]. According to the results of present study, the regulation of gene expression to submergence adaptation is essential for formation of storage organ, such as *ERF*, *ADH*, *MYB*, SNF like protein gene, SOS2 like protein kinase gene, SNF-related kinase gene, superoxide dismutase gene, G-box binding protein gene, and heat shock protein gene [45]. *ADH* (alcohol dehydrogenase) plays an important role in low-oxygen tolerance. Expression and activity of ADH are thought to be an indicator of oxygen limitation [48], [49], [50], [51], [52], [53]. Therefore, ADH enhanced their expression during rhizome development which is essential for lotus root to adapt to submergence condition.

Ethylene not only mediates a range of different biotic and abiotic stress responses 54. but is also involved in storage organ development and submergence adaptation [55]. It is considered to be a critical hormone in ethylene-mediated growth promotion which also helps to confront anoxia [56]. The signal transduction to oxygen shortage during stem development is dependent on ethylene, and elongation of stem in response to ethylene is a widely spread adaptation to anaerobic stress [56]. Ethylene responsive element binding factor (ERF) belongs to AP2 which regulate plant metabolism process. Many ERF genes were found to be responsive to abiotic stresses such as cold, wounding or drought stress, and also induced by some kinds of hormones such as ethylene, JA, and SA [57]. In four developmental stages of lotus root, we also observed enhanced mRNA level of several *ERFs* during development of storage organ, which indicates that regulations of ethylene also affects the growth and development of lotus root. In Arabidopsis, *ERFs* have been studied and their functions were also identified in recent years [58]. Three ethylene response-factor (ERF) genes were identified to be located on an *sus1* locus, which was considered as a submergence tolerance QTL in rice [59], [60], [61]. In this study, we found increase in the transcriptional level of many members of ERF family during growth of lotus root, suggesting that *ERFs* also aid to lotus root survival in anaerobic stress.

It was reported that energy regulation was a measure to alleviate the hurt acquired because of submergence, and at the same time, many critical processes for storage organ formation also need energy consumption [62], [63], [64], [65]. ATP is normally generated from glycolysis in plant cells to sustain plant growth. For generation of ATP, ATP/ADP translocator (L23655) is thought to be a gated pore through which ADP and ATP are exchanged between the mitochondrial matrix and the cytoplasm. ATP/ADP translocator is an inner membrane protein which is observed to enhance the number and morphology of storage organ in transgenic plants. For lotus root, an ATP/ADP translocator was observed to improve the transcriptional level during rhizome development, suggesting that enhanced ATP level is helpful for survival and plant growth [66]. Evidence shows that delivery of NADPH/NADH reduced ferredoxin, and ATP in the specific cellular compartments occurs for energy-consuming reactions under stress condition. Generally, NADPH/NADH, and reduced ferredoxin are not directly transported across bio-membranes, therefore, indirect transport of reducing equivalents is achieved by malate/oxaloacetate shuttles, involving malate dehydrogenase (MDH) and NADP-MDH (Isoenzyme of MDH) for the interconversion of oxaloacetic acid and malate [67], [68]. MDH is one of the more active enzymes in peroxisomes, mitochondria, chloroplast, glyoxysomes, and cytoplasm. The activities of the enzymes of malate and NADP-MDH valves are changed when plants are subjected to conditions such as high light, high CO2, nutrition or stress. A lot of evidences show that NADP-MDH is involved in response to environmental factors. When plants are subjected to stress conditions, which require changed activities of the enzymes of malate valves, changed expression levels of MDH isoforms can be observed [69], [70], [71], [72] . The expression of ATP/ADP translocator (L23655), MDH (L8940) and NADP-MDH (L26121) was enhanced in L1/L2, L2/L3, and L3/L4 libraries. Therefore, these genes might be an adaptive response for lotus root in submergence conditions during storage development.

### Carbohydrate/storage Metabolism

Starch is considered as an important component in the storage organ (rhizome, corm, tuber, and bulb). Simultaneous swelling of storage organ and accumulation of starch have already been testified [73], suggesting that synthesis of starch shows high coordination with formation of storage organ [74]. In our study, a gene encoding granule-bound starch synthase was found to enhance mRNA level during the rhizome development (Table 4). Granule-bound starch synthase is believed to have a higher amounts of starch synthesized in plant storage organs [75]. It is similar to the report of Hanashiro et al. (2008) [76]. in this study, *GBSS* enhanced the transcriptional level in L1/L2 and L2/L3 libraries, which indicates that the expression profile of *GBSS* has high correlation with the development of rhizome.

Patatin, which is identical to glycoprotein, is usually believed as a major storage protein in rhizome, tuber, corm, and bulb. We found that expression of *patatin* (L10302) decreased in L1/L2 libraries and L2/L3 libraries, but enhanced in L3/L4 libraries, suggesting that patatin accumulation increases in the later swelling stage. Compared with other storage proteins, patatin is more stable because no degradation products are detected during tuber development. Evidence shows that *patatin* might be involved in metabolism because acyl hydrolase has been found to be encoded by this gene [77], [78]. Another study shows that *patatin* is involved in pollen development [79], [80], which is further testified by Vancanneyt et al. 81. Further study shows that patatin is associated with early events of formation of underground storage organ according to its expression profile [82], [83]. In stolon tips of non-induced plants, only a small amount of patatin is found, which rapidly accumulates during storage organ formation [84]. Accumulation of patatin has high correlation with the swelling of storage organ because it is observed to be synthesized only in stolon and storage organ and induced by sucrose [85], [86], [87]. For lotus root, *patatin* enhances the expression only in L3/L4 libraries, which obviously showed that *patatin* was accumulated in later swelling stages of rhizome. High expression *pattern* might have some relationship with rhizome development, although the data of present result did not show any correlation between patatin and formation of storage organ.

Sugar can provide enough materials and energy for plant to complete some important activities during its life cycle [88]. The processes of sugar synthesis, transport, consumption, and storage have been widely studied in the past decades [89]. Evidence shows that soluble carbohydrates, most notably sucrose are considered to be the strong inducers of underground storage organ formation, because increasing concentration of sucrose in medium during cultivation leads to higher numbers of tubers [90]. Sucrose plays its role as an inducing signal molecule, and enhancing the level of sucrose in stolons results in an increased number of initiated storage organs [91]. A gene (L2349) encoding sucrose improved its transcriptional level in L1/L2 libraries (Table 4), suggesting that accumulation of sucrose is helpful for rhizome formation of lotus root. Further evidence also shows that SNF1 kinase (the sucrose non-fermenting-1) is involved in sugar-signaling pathways to regulate metabolism of carbohydrate or other storage proteins [92]. mRNA level of SNF1of lotus root was enhanced in L1/L2 (log_2_ ratio (L2/L1: 1.2) and L2/L3 (log_2_ ratio (L2/L1: 6.39) and L3/L4 (log_2_ Ratio (L2/L1: 2.08) libraries. From the characteristics of gene expression, SNF1 (L10886) undoubtedly promoted the formation and development of rhizome (Table S1).

### Up-regulation of Transcription Factors during Rhizome Formation

Transcription factors regulate gene expression during cell metabolism processes. Transcriptional levels of 14, 20, and 20 regulators were enhanced in L1/L2, L2/L3, and L3/C4 libraries, respectively (Table 5). For these transcription factors, we found that CaM-binding protein (L1140) and AP2 domain class transcription factors (L1140) have been identified to perform critical roles in the formation of underground storage organ. Ca^2+^ has been testified to perceive endogenous and exogenous signals as a second messenger before system responses [93]. Calmodulin is a sensor of Ca^2+^, and many cellular processes are modulated after Ca^2+^ binds to CaM [94]. Ca^2+^/CaM have already been identified to be involved in the formation of storage organs [95]. Further study shows that transgenic potato plants with constitutive expression of a CaM gene (*PCM1*) produces more elongated tuber [96]. CaM-binding proteins have been found in many plants and their functions have also been considered for the development of storage organs [93], [97].

A potato CaM-binding protein (PCBP) is found to play an important role in signal transduction during tuber formation according to characteristics of its expression [98]. We observed that calmodulin-like protein (L16799) and calmodulin binding protein (L22245) have similar expression profiles in L1/L2, L2/L3, and L3/L4 libraries. From stolon to initial swelling stage of rhizome, expression levels of these two genes improved, and decreased from initial swelling stage to middle swelling stage and middle swelling stage to swelling stage, indicating that Ca^2+^/CaM might be involved in processes from stolon stage to initial swelling stage of rhizome.

In this study, the expression level of ethylene responsive factor was found to be enhanced (Table 5). From the characteristics of expression, we could conclude that this gene play an important role in rhizome formation. Ethylene is not only involved in a range of biotic and abiotic stress responses, especially to help wetland plants to adapt to anaerobic condition, but also mediates swelling of underground storage organs [99]. At the same time, AP2 domain class transcription factor, which is unique in plant kingdom also shows multi-functions in metabolism from response to stresses to regulation of plant development [100]. Overexpression of an AP2 domain class transcription factor, confers potato plant more resistance to abiotic stress, whereas down-regulation leads to a series of other effects such as decreased cell size, plant height, hypocotyl elongation and fertility [101]. Several AP2 domain class transcription factors are found to control flowering time [102]. It is reported that a putative AP2 domain class transcription factor (*WRI1*), regulates the storage metabolism in Arabidopsis seed. Constitutive expression of this gene leads to improvement of the seed oil content and triacylglycerols [103]. Transgenic rice plant with an AP2 domain class transcription factor increases the expression of waxy gene, which directly results in a change of storage content [104]. In addition, AP2 domain class transcription factors have been found to be induced by abscisic acid (ABA) and sugar, suggesting that this gene might be involved in ABA and sugar signal transduction pathway [105]. Therefore, future studies about role of AP2 domain class transcription factor in rhizome formation should be carried out with molecular engineering techniques.

### Hormonal Regulation

Swelling of underground storage organ is affected by various environmental and endogenous factors. Short photoperiods, low temperatures, low nitrogen, and hormone favor the formation of storage organ [90]. In literature, many reports describe the importance of gibberellic acid (GA), JA, ethylene for the formation of storage organ. GA content is enhanced in transgenic plants by overexpression of GA oxidase gene, and elevation of GA content leads to transgenic potato plants that require a longer duration of short-day photoperiods to form storage organ. Whereas, inhibition of this enzyme activity results in earlier formation of these organs as compared with that of non transgenic plants [27]. Decrease in GA content in a dwarf mutant of *S. tuberosum* ssp. *Andigena* showed lead to formation of storage organ both in LD and SD condition. However, the storage organ is also inhibited in SD when GA biosynthesis is inhibited [9]. These results suggest that GA is probably involved in the photoperiodic induction to regulate the formation of storage organ. In addition, Xu et al. 106. observed that high content of GA promotes stolon elongation and inhibit storage organ formation. In this study, the expression of GA 20-oxidase gene (L18298) involved in GA biosynthesis pathway is high in L1 libraries (stolon stage), whereas it decreased in L2 and L3, but increased in L4 stages (data not shown). Enhancing GA level in stolon probably benefited the elongation of stolon and decreased GA level in L2 and L3 might have benefited the rhizome development. As we all known, in later development stages of lotus root, few new rhizomes will be formed again, and this is the reason why we observed increase in the expression of gibberellin 20 oxidase in the later swelling stage.

JA is widely distributed in plants and exerts a critical function in plant development and growth [107]. Furthermore, JA also plays a significant role in stress response [108]. Several studies have been performed to study the effects of JA on the swell of underground storage organ. Endogenous JA is believed to be a strong inducer of storage organ in dicot potato plants and monocot yam plants [109]. Ravnikar et al (1993) reports that exogenous JA plays an important role in the formation of garlic bulbs [110]. Zeal 111. found that application of exogenous JA not only induces the formation of storage organ, but simultaneously enhances the storage organ number. In addition, tuber formation of *Pterostylis sanguinea* is also promoted by exogenous JA [112]. In the four stages of rhizome development, we found that many JA-induced genes (MYB, transcription factor bHLH, bZIP, and AP2/ERF domain-containing transcription factor, lipoxygenase) enhanced transcriptional level during rhizome development [113], [114], [115], [116], [117]. These JA-induced genes, which promote storage organ formation, have already been testified in the past decades. Therefore, JA and JA responsive genes are helpful for rhizome formation. In addition, some storage organ-related genes were also documented in this study (Table 6), and the relationship between gene expression and storage organ formation has been testified in literature [118], [119], [120], [121], [122], [123]. Overall, expression profiles of these genes show that formation of rhizome is very complex and regulated by multiple genes.

### Conclusions

Using high-throughput tag-sequencing (based on Solexa Genome Analyzer Platform), four expression libraries were constructed and analyzed from different developmental stages for rhizome formation. Based on the results of tag-sequencing, after comparison with the existing databases, overall 20471, 18785, 23448, and 21778 tags were annotated from four developmental stages (stolon stage, initial swelling stage, middle swelling stage, and swelling stage) respectively. In addition, several DEGs relevant to rhizome formation were also identified from these four libraries. Expression of nine genes was studied by qRT-PCR to verify the results of tag-sequencing. The qRT-PCR results revealed that gene expression showed high correlation with the tag sequencing analysis.

## Supporting Information

Figure S1 Developmental stages of lotus rhizome including stolon stage, initial stage, middle swelling stage and later swelling stage.
(TIF)

Figure S2 Sequencing saturation analysis of three libraries. L1: tag-sequencing for stolon stage; L2: tag-sequencing for initial swelling stage; L3: tag-sequencing for middle swelling stage; L4: tag-sequencing for later swelling stage.
(TIF)

Figure S3 GO analysis of genes expressed during the rhizome formation. All the genes identified in L1/L2, L2/L3, and L3/L4 libraries were classified into 26 classifications according to gene functions.
(TIF)

Figure S4 Expression profiles of 18 genes at stolon stage, initial swelling stage and middle swelling stage in rhizome lotus, flower lotus, and seed lotus.
(TIF)

Table S1 Genes were found to enhance their expression in L1/L2, L2/L3, and L3/L4 libraries. These genes were listed in descending order according to alteration of expression during rhizome formation.
(XLS)

## References

[1] Xue JH, Dong WP, Chen T, Zhou SL (2012) Nelumbonaceae: Systematic position and species diversification revealed by the complete chloroplast genome. of Systematics and Evolution50: 477–487.

[2] Sakamoto Y (1977) Lotus. Tokyo: Hosei University Press [in Japanese].

[3] Liu J, Zhang M, Wang S (2010) Processing characteristics and flavour of full lotus root powder beverage. *J Sci* Food Agric 90: 2482–2489. 20737547 10.1002/jsfa.4110

[4] Slocum PD, Robinson P (1996). Water gardening, water lilies and lotuses. Portland, OR: Timber Press.

[5] Borgi W, Ghedira K, Chouchane N (2007) Antiinflammatory and analgesic activities of zizyphus lotus root barks. Fitoterapia. 78: 16–19. 10.1016/j.fitote.2006.09.010 17107758

[6] Renato BRAZ, Hechenleitner AAW, Cavalcanti OA (2007) Extraction, structural modification and characterization of lotus roots polysaccharides (*Nelumbo nucifera* Gaertn). Excipient with potential application in modified drug delivery systems. Lat. Am. J. Pharm 26: 706–710.

[7] Terashima M, Awano K, Honda Y, Yoshino N, Mori T, et al.. (2011) “Arteries within the artery” after kawasaki diease-A lotus root appearance by intravascular ultrasound. Circulation DOI: 10.1161/01.CIR.0000030708.86783.92 10.1161/01.cir.0000030708.86783.92 12176965

[8] Vartapetian BB, Jackson M (1997) Plant adaptations to anaerobic stress. Ann of Bot 79: 3–20.

[9] Vreugdenhil D, Struik PC (1989) An integrated view of the hormonal regulation of tuber formation in potato (*Solanum tuberosum*). Physiol Plant 75: 525–531.

[10] Hannapel DJ (1991) Characterization of the early events of potato tuber development. Physiol. Plant 83: 568–573.

[11] MacLeod RAF, Dirks WG, Matsuo Y, Kaufmann M, Milch H, et al (1999) Widespread intraspecies cross-contamination of human tumor cell lines arising at source. Int J Cancer. 83: 555–563. 10.1002/(sici)1097-0215(19991112)83:4<555::aid-ijc19>3.0.co;2-2 10508494

[12] Paiva E, Lister RM, Park WD (1983) Induction and accumulation of major tuber proteins of potato in stems and petioles. Plant Physiol 71: 161–168. 16662778 10.1104/pp.71.1.161 PMC1066005

[13] Park WM (1983) Tuber proteins of potato- a new and surprising molecular system. Plant Mol Biol Rep 1: 61–66.

[14] Masuda JI, Ozaki YK, ·Okubo HS (2007) Rhizome transition to storage organ is under phytochrome control in lotus *(Nelumbo nucifera).* . Planta 226: 909–915. 17520280 10.1007/s00425-007-0536-9

[15] Jackson SD (1999) Multiple signaling pathways control tuber induction in potato. Plant Physiol 119: 1–8. 9880339 10.1104/pp.119.1.1 PMC1539201

[16] Inui H, Ogura Y, Kiyosue T (2010) Overexpression of Arabidopsis thaliana LOV KELCH REPEAT PROTEIN 2 promotes tuberization in potato (Solanum tuberosum cv. May Queen). FEBS Lett. 584: 2393–2396. 10.1016/j.febslet.2010.04.031 20399775

[17] Martinez-Garcia JF, Virgos-Soler A, Prat S (2002) Control of photoperiod-regulated tuberization in potato by the Arabidopsis flowering-time gene *constans* . PNAS 99: 15211–15216. 12393812 10.1073/pnas.222390599 PMC137569

[18] Kim WY, Fujiwara S, Suh SS, Kim J, Kim Y, et al (2007) ZEITLUPE is a circadian photoreceptor stabilized by GIGANTEA in blue light. Nat 449: 356–360. 10.1038/nature06132 17704763

[19] Chen H, Banerjee AK, Hannnapel DJ (2004) The tandem complex of BEL and Knox partners is required for transcriptional repression of *ga20ox1*. Plant J. 38: 276–284. 10.1111/j.1365-313X.2004.02048.x 15078330

[20] Abelenda JA, Navarro C, Prat S (2011) From the model to the crop: genes controlling tuber formation in potato. Cur Opin in Biotech 22: 287–292. 10.1016/j.copbio.2010.11.013 21168321

[21] Jackson S, Heyer A, Dietze J, Prat S (1996) Phytochrome B mediates the photoperiodic control of tuber formation in potato. Plant J 9: 159–166.

[22] Alisdair RF, Willmitzer L (2001) Molecular and biochemical triggers of potato. Plant Physiol 127: 1459–1465. 11743089 PMC1540178

[23] Appeldoorn NJG, de Bruijn SM, Koot-Gronsveld EAM, Visser RGF, Vreugdenhil D, et al (1997) Developmental changes of enzymes involved in conversion of sucrose to hexose-phosphate during early tuberization of potato. Planta 202: 220–226.

[24] Jasik J, de Klerk GJ (2006) Effect of methyl jasmonate on morphology and dormancy development in lily bulblets regenerated *in vitro* . J Plant Growth Regu 25: 45–51.

[25] Kim KJ, Kim KS (2005) Changes of endogenous growth substances during bulb maturation after flowering in *Lilium* oriental hybrid ‘Casa Blanca’ Acta Hort. 570: 661–667.

[26] Usha PR, Rajasekaran RL, Claude DC, Samuel KA, Kevin JS (2011) Role of ethylene and jasmonic acid on rhizome induction and growth in rhubarb (Rheum rhabarbarum L.). Plant Cell Tiss Organ Cult 105: 253–263.

[27] Carrera E, Bou J, Garcia-Martinez JL, Prat S (2000) Changes in GA 20-oxidase gene expression strongly affect stem length, tuber induction and tuber yield of potato plants. Plant J 22: 247–256. 10849342 10.1046/j.1365-313x.2000.00736.x

[28] Galis I, Macas J, Vlasak J, Ondrej M, van Onckelen HA (1995) The effect of an elevated cytokinin level using the ipt gene and N-6 benzyladenine on a single node and intact potato plant tuberization *in vitro* . J Plant Growth Regul 14: 143–150.

[29] Bhat SR, Chandel KPS, Kacker A (1994) *In vitro* induction of rhizomes in zinger (*Zingiber officinale* Rosc.). Indian J Expt Biol 32: 340–344.

[30] Vreugdenhil D, Bindels P, Reinhoud P, Klocek J, Hendriks T (1994) Use of the growth retardant tetcyclacis for potato tuber formation *in vitro.* . J Plant Growth Regul 14: 257–265.

[31] himann KV, Skoog F (1934) On the inhibition of bud development and other functions of growth substance in *Vicia faba*. Proc. R. Soc. Lond. B Biol. Sci 114: 317–339.

[32] Abeles FB, Morgan PW, Saltveit Jr ME (1992) Ethylene in Plant Biology. San Diego: Academic Press.

[33] Neuteboom CE, Lada RL, Caldwell CD, Eaton L, Havard P (2002) Ethephon and spermidine enhance bulking in dicer carrots (Daucus carota var. sativus). PGRSAQ 30: 77.

[34] Hu FY, Wang D, Zhao XQ, Zhang T, Sun HX, et al (2011) Identification of rhizome-specific genes by genome-wide differential expression Analysis in Oryza longistaminata. BMC Plant Biology 11: 18. 21261937 10.1186/1471-2229-11-18 PMC3036607

[35] Mingo-Castel AM, Smith OE, Kumamoto J (1976) Studies on the carbon dioxide promotion and ethylene inhibition of tuberizaton in potato explants cultured *in vitro* . Plant Physiol 57: 480–485. 16659510 10.1104/pp.57.4.480 PMC542056

[36] Bentley DR (2006) Whole-genome re-sequencing. Curr Opin Genet Dev 16: 545–552. 17055251 10.1016/j.gde.2006.10.009

[37] Ansorge WJ (2009) Next-generation DNA sequencing techniques. New Biotechnol 25: 195–203. 10.1016/j.nbt.2008.12.009 19429539

[38] Qi XH, Xu XW, Lin XJ, Zhang WJ, Chen XH (2011) Identification of differentially expressed genes in cucumber (Cucumis sativus L.) root under waterlogging stress by digital gene expression profile. Genomics. doi:10.1016/j.ygeno 2011.12.008 10.1016/j.ygeno.2011.12.008 22240004

[39] Wang QQ, Liu F, Chen XS, Ma XJ, Zeng HQ, et al (2010) Transcriptome profiling of early developing cotton fiber by deep-sequencing reveals significantly differential expression of genes in a fuzzless/lintless mutant. Genomics 96: 369–376. 20828606 10.1016/j.ygeno.2010.08.009

[40] Mach J (2011) Unpureeing the tomato layers of information revealed by microdissection and high-throughput transcriptome sequencing. Plant Cell. doi: 10. 1105/tpc 111.231111 10.1105/tpc.111.231111 PMC3246339 22076842

[41] Wang FD, Li LB, Li HY, Liu LF, Zhang YH, et al.. (2012) Transcriptome analysis of rosette and folding leaves in Chinese cabbage using high-throughput RNA sequencing. Genomics. doi: 10.1016/j.ygeno.02.005 10.1016/j.ygeno.2012.02.005 22387604

[42] Wu T, Qin ZW, Zhou XY, Feng Z, Du YL (2010) Transcriptome profile analysis of floral sex determination in cucumber. J Plant Physiol 167: 905–913. 20303197 10.1016/j.jplph.2010.02.004

[43] Perata P, Alpi A (1993) Plant responses to anaerobiosis. Plant Sci. 93: 1–7.

[44] Subbaiah CC, Zhang J, Sachs MM (1994) Involvement of intracellular calcium in anaerobic gene expression and survival of maize seeding. Plant Physiolo 105: 369–376. 10.1104/pp.105.1.369 PMC159365 7518090

[45] Vartapetian BB, Jackson MB (1997) Plant adaptations to anaerobic stress. Ann. Bot 79: 3–30.

[46] Ishizawa A, Murakami S, Kawakami Y, Kuramochi H (1999) Growth and energy status of arrowhead tubers, pondweed turions and rice seeding under anoxic conditions. Plant Cell Environ. 22: 505–514.

[47] Crawford RMM, Braendle R (1996) Oxygen deprivation stress in a changing environment. J Exp Bot 47: 145–159.

[48] Jung SH, Lee JY, Lee DH (2003) Use of SAGE technology to reveal changes in gene expression in Arabidopsis leaves undergoing cold stress. Plant Mol Biol 52: 553–567. 12956526 10.1023/a:1024866716987

[49] Shiao TL, Ellis MH, Dolferus R, Dennis ES, Doran PM (2002) Overexpression of alcohol dehydrogenase or pyruvate decarboxylase improves growth of hairy roots at reduced oxygen concentrations. Biotechnol Bioeng 77: 455–461. 11787018 10.1002/bit.10147

[50] Gibbs J, Greenway H (2003) Mechanisms of anoxia tolerance in plants. I. Growth, survival and anaerobic catabolism. Funct. Plant Biol 30: 1–47. 10.1071/PP98095 32688990

[51] Baxter-Burrell A, Chang R, Springer P, Bailey-Serres J (2003) Gene and enhancer traptransposable elements reveal oxygen deprivation-regulated genes and their complex patterns of expression in *Arabidopsis*. Ann. Bot 91: 129–41. 10.1093/aob/mcf119 PMC4244989 12509334

[52] Xie ZD, Li DM, Wang LJ, Sack FD, Grotewold E (2010) Role of the stomatal development regulators FLP/MYB88 in abiotic stress responses. Plant J 64: 731–739. 21105921 10.1111/j.1365-313X.2010.04364.x

[53] Hoeren FU, Dolferus R, Wu Y, Peacock WJ, Dennis ES (1998) Evidence for a role for AtMYB2 in the induction of the *Arabidopsis* alcohol dehydrogenase gene (*ADH1*) by low oxygen. Genetics 149: 479–490. 9611167 10.1093/genetics/149.2.479 PMC1460183

[54] Grichko VP, Glick BR (2001) Ethylene and flooding stress in plants. Plant Physiol. Biochem 39: 1–9.

[55] Suge H, Kusanagi T (1975) Ethylene and carbon dioxide: regulation of growth in two perennial aquatic plants, arrowhead and pondweed. Plant and Cell Physiol 16: 65–72.

[56] Jackson MB (2008) Ethylene-promoted Elongation: an Adaptation to Submergence Stress. Ann. Bot 101: 229–248. 10.1093/aob/mcm237 PMC2711016 17956854

[57] Singh KB, Foley RC, Sánchez LO (2002) Transcription factors in plant defense and stress responses. Cur Opin in Plant Biol 5: 430–436. 10.1016/s1369-5266(02)00289-3 12183182

[58] Fujimoto SY, Ohta M, Usui A, Shinshi H, Ohme-Takagi M (2000) *Arabidopsis* ethylene-responsive element binding factors act as transcriptional activators or repressors of GCC box-mediated gene expression. Plant Cell 12: 393–404. 10715325 10.1105/tpc.12.3.393 PMC139839

[59] Vij S, Tyagi AK (2007) Emerging trends in the functional genomics of the abiotic stress response in crop plants. Plant Biotech J (5) 361–380. 10.1111/j.1467-7652.2007.00239.x 17430544

[60] Fukao T, Xu K, Ronald PC, Bailey-Serres J (2006) A Variable Cluster of Ethylene Response Factor–Like Genes Regulates Metabolic and Developmental Acclimation Responses to Submergence in Rice. 18: 2021–2034. 10.1105/tpc.106.043000 PMC1533987 16816135

[61] Zhou SF, lü SH 2, Fu FL, Lan H, Zhang ZM, et al (2011) Over-expression of *Sub1A*, a submergence tolerance gene from rice, confers enhanced hypoxic stress tolerance in transgenic tobacco plants African J Biotech. 10: 17934–17939.

[62] Bailey-Serres J, Voesenek L ACJ (2008) Flooding stress: acclimations and genetic diversity. Annu Rev Plant Biol 59: 313–339. 18444902 10.1146/annurev.arplant.59.032607.092752

[63] Pederson O, Rich SM, Colmer TD (2009) Surviving floods: leaf gas films improve O2 and CO2 exchange, root aeration, and growth of completely submerged rice. Plant J 58: 147–156. 19077169 10.1111/j.1365-313X.2008.03769.x

[64] Rocha M, Licausi F, Araujo WL, Nunes-Nesi A, Sodek L, et al (2010) Glycolysis and the tricarboxylic acid cycle are linked by alanine aminotransferase during hypoxia induced by waterlogging of lotus japonicus. Plant Physiol 152: 1501–1513. 20089769 10.1104/pp.109.150045 PMC2832266

[65] Geigenberger P, Stitt M, Fernie AR (2004) Metabolic control analysis and regulation of the conversion of sucrose to starch in growing potato tubers. Plant Cell Environ 27: 655–673.

[66] Tjaden J, Mohlmann T, Kampfenkel K, Henrichs G, Neuhaus HE (1998) Altered plastidic ATP/ADP-transporter activity influences potato tuber morphology, yield and composition of starch. Plant J 16: 531–540.

[67] Gietl C (1992) Malate dehydrogenase isoenzymes: cellular locations and role in the flow of metabolites between the cytoplasm and cell organelles. Biochem Biophys Acta 1100: 217–234. 1610875 10.1016/0167-4838(92)90476-t

[68] Scheibe R (2004) Malate valves to balance cellular energy supply. Physiol Plant 120: 21–26. 15032873 10.1111/j.0031-9317.2004.0222.x

[69] Hameister S, Becker B, Holtgreffe S, Strodtkotter I, Linke V, et al (2007) Transcriptional regulation of the NADP dependent malate dehydrogenase: comparative genetics and identification of DNA-binding proteins. J Mol Evol 65: 437–455. 17925997 10.1007/s00239-007-9025-9

[70] Becker B, Holtgrefe S, Jung S, Wunrau C, Kandlbinder A, et al (2006) Influence of the photoperiod on redox regulation and stress responses in Arabidopsis thaliana L. (Heynh.) plants under long- and short-day conditions. Planta 224: 380–393. 16435132 10.1007/s00425-006-0222-3

[71] Cushman JC (1993) Molecular cloning and expression of chloroplast NADP-malate dehydrogenase during Crassulacean acid metabolism induction by salt stress. Photosynth Res 35: 15–27. 24318617 10.1007/BF02185408

[72] Sahi C, Singh A, Kumar K, Blumwald E, Grover A (2006) Salt stress response in rice: genetics, molecular bilogy, and comparative genomics. Func Inter Genomics 6: 263–284. 10.1007/s10142-006-0032-5 16819623

[73] Burton WG, Van Es A, Hartmans KJ (1992) The physics and physiology of storage. The Potato Crop 2: 608–727.

[74] Geigenberger P, Merlo L, Reimholz R, Stitt M (1994) When growing potato tubers are detached from their mother plant there is a rapid inhibition of starch synthesis, involving inhibition of ADP-glucose pyrophosphorylase. Planta 193: 486–493.

[75] Nakamura T, Vrinten P, Hayakawa K, Ikeda J (1998) Characterization of a Granule-Bound Starch Synthase Isoform Found in the Pericarp of Wheat. Plant Physiol. 118: 451–459. 10.1104/pp.118.2.451 PMC34820 9765530

[76] Hanashiro I, Itoh K, Kuratomi Y, Yamazaki M, Igarashi T, et al (2008) Granule-Bound Starch Synthase I is Responsible for Biosynthesis of Extra-Long Unit Chains of Amylopectin in Rice. Plant Cell Physiol 49: 925–933. 18430767 10.1093/pcp/pcn066

[77] Racusen D (1985) Patatin and esterase in desire potato tuber. Food Ind 9: 61–367.

[78] Andrews DL. Beames B, Summers MD, Park WD (1988) Characterisation of the lipid acyl hydrolase activity of the major potato (Solanum tuberosum) tuber protein, patatin, by cloning and abundant expression in a baculovirus vector. Biochem J 252: 199–206. 3048246 10.1042/bj2520199 PMC1149124

[79] Sawhney VK, Nave EB (1986) Enzymatic changes in post-meiotic anther development in Petunia hybrida. 11. Histochemical localisation of esterase, peroxidase, malate- and alcohol dehydrogenase. J Plant Physiol 125: 467–473.

[80] Delvallee I, Dumas C (1988) Anther development in Zea *mays* : Changes in protein, peroxidase, and esterase patterns. J Plant Physiol 132: 210–217.

[81] Vancanneyt G, Sonnewald U, Hofgen R, Willmitzer (1989) Expression of a patatin-like protein in the anthers of potato and sweet pepper flowers. Plant Cell 533–540. 10.1105/tpc.1.5.533 PMC159787 12359901

[82] Perl A, Aviv D, Willmitzer L, Galun E (1991) In vitro tuberization in transgenic potatoes harboring 13-glucuronidase linked to a patatin promoter: effects of sucrose levels and photoperiods. Plant Sci 73: 87–95.

[83] Bamfalvi Z, Kostyal Z, Barta E (1994) *Solanum brevidens* possesses a non-sucrose-inducible patatin gene. Mol Gen Genet 245: 517–522. 7808402 10.1007/BF00302265

[84] Hannapel DJ, Miller J C, Park WD (1985) Regulation of Potato Tuber Protein Accumulation by Gibberellic Acid. Plant Physiol 78: 700–703. 16664311 10.1104/pp.78.4.700 PMC1064807

[85] Stupar RM, Beaubien KA, Jin WW, Song JQ, Lee MK, et al (2006) Structural Diversity and Differential Transcription of the Patatin Multicopy Gene Family During Potato Tuber Development. Genetics 172: 1263–1275. 16322504 10.1534/genetics.105.051219 PMC1456224

[86] Rocha-Sosa M, Sonnewald U, Frommer W, Stratmann M, Schell J (1989) Both developmental and metabolic signals activate the promoter of a class I patatin gene. EMBO J 8: 23–29. 16453867 10.1002/j.1460-2075.1989.tb03344.x PMC400768

[87] Grierson C, Du JS, de Torres ZM, Beggs K, Smith C (1994) Separate cis sequences and trans factors direct metabolic and developmental regulation of a potato tuber storage protein gene. Plant J 5: 815–826. 8054988 10.1046/j.1365-313x.1994.5060815.x

[88] Gibson SI (2005) Control of plant development and gene expression by sugar signaling. Cur Opin in Plant Biol 8: 93–102. 10.1016/j.pbi.2004.11.003 15653406

[89] Sheen J, Zhou L, Jang JC (1999) Sugars as signaling molecules. Cur Opin in Plant Biol 2: 410–418. 10.1016/s1369-5266(99)00014-x 10508760

[90] Fernie AR, Willmitzer L (2001) Molecular and biochemical triggers of potato tuber development. Plant Physiol 127: 1459–1465. 11743089 PMC1540178

[91] Dijkwel PP, Huijser C, Weisbeek PJ, Chua NH, Smeekens SCM (1997) Sucrose control of phytochrome A signaling in *Arabidopsis* . Plant Cell 9: 583–595. 9144963 10.1105/tpc.9.4.583 PMC156941

[92] Tiessen A, Prescha K, Branscheid A, Palacios N, Mckibbin R, et al (2003) Evidence that SNF1-related kinase and hexokinase are involved in separate sugar-signaling pathways modulating post-translational redox activation of ADP-glucose pyrophosphorylase in potato tubers. Plant J 35: 490–500. 12904211 10.1046/j.1365-313x.2003.01823.x

[93] Kim MC, Chung WS, Yun DJ, Cho M J (2009) Calcium and calmodulin-mediated regulation of gene expression in plants. Mol Plant 2: 13–21. 19529824 10.1093/mp/ssn091 PMC2639735

[94] Hoeflich KP, Ikura M (2002) Calmodulin in action: diversity in target recognition and activation mechanisms. Cell 108: 739–742. 11955428 10.1016/s0092-8674(02)00682-7

[95] Balamani V, Veluthambi K, Poovaiah BW (1986) Effect of calcium on tuberization in potato (*Solanum tuberosum* L.). Plant Physiol 80: 856–858. 16664731 10.1104/pp.80.4.856 PMC1075219

[96] Poovaiah BW, Takezawa D, An G, Han TJ (1996) Regulated expression of a calmodulin isoform alters growth and development in potato. J. Plant Physiol 149: 553–558. 11540589 10.1016/s0176-1617(96)80333-6

[97] Bouché N, Scharlat A, Snedden W, Bouchez D, Fromm H (2002) A novel family of calmodulin-binding transcription activators in multicellular organisms. J Biol Chem 277: 21851–21861. 11925432 10.1074/jbc.M200268200

[98] Reddy ASN, Safadi F, Narasimhulu SB, Golovkin M, Hu X (1996) Isolation of a novel calmodulin-binding protein from *Arabidopsis tha/iana.* . J Biol Chem 271: 7052–7060. 8636137 10.1074/jbc.271.12.7052

[99] Suge H, Kusanagi T (1975) Ethylene and carbon dioxide: regulation of growth in two perennial aquatic plants, arrowhead and pondweed. Plant Cell Physiol 16, 65–72.

[100] Krizek BA (2009) Aintegumenta and aintegumentalke6 act redundantly to regulate Arabidopsis floral growth and patterning. Plant Physiol 150: 1916–1929. 19542297 10.1104/pp.109.141119 PMC2719149

[101] Dietz KJ, Vogel MO, Viehhauser A (2010) AP2/EREBP transcription factors are part of gene regulatory networks and integrate metabolic, hormonal and environmental signals in stress acclimation and retrograde signaling. Protoplasma 245: 3–14. 20411284 10.1007/s00709-010-0142-8

[102] Feng JX, Liu D, Pan Y, Gong W, Ma LG, et al (2005) An annotation update via cDNA sequence analysis and comprehensive profiling of developmental, hormonal or environmental responsiveness of the Arabidopsis AP2/EREBP transcription factor gene family. Plant Mol Biol 59: 853–868. 16307362 10.1007/s11103-005-1511-0

[103] Cernac A, Benning C (2004) WRINKLED1 encodes an AP2/EREB domain protein involved in the control of storage compound biosynthesis in Arabidopsis. Plant J 40: 575–585. 15500472 10.1111/j.1365-313X.2004.02235.x

[104] Zhu Y, Cai XL, Wang ZY, Hong MM (2003) An interaction between a MYC protein and an EREBP protein is involved in transcriptional regulation of the rice Wax gene. J Biol Chem 278: 47803–47811. 12947109 10.1074/jbc.M302806200

[105] Rook F, Corke F, Card R, Munz G, Smith C, et al (2001) Impaired Suc-induction mutants reveal the modulation of sugar-induced starch biosynthetic gene expression by abscisic acid signalling. Plant J 26: 421–433. 11439129 10.1046/j.1365-313x.2001.2641043.x

[106] Xu X, van Lammeren AAM, Vermeer E, Vreugdenhil D (1998) The role of gibberellin, abscisic acid, and sucrose in the regulation of potato tuber formation in vitro. Plant Physiol 117: 575–584. 9625710 10.1104/pp.117.2.575 PMC34977

[107] Sembdner G, Parthier B (1993) The biochemistry and the physiological and molecular actions of jasmonates. Annu. Rev. Plant Physiol 44: 569–589.

[108] Creelman RA, Mullet JE (1997) Biosynthesis and action of jasmonates in plants. Annu. Rev. Plant Physiol. 48: 355–381. 10.1146/annurev.arplant.48.1.355 15012267

[109] Koda Y (1992) The role ofjasmonic acid and related compounds in the regulation of plant development. Int Rev Cytol 135: 155–199. 1618607 10.1016/s0074-7696(08)62040-9

[110] Ravnikar M, Zel J, Plaper I, Spacapan A (1993) Jasmonic Acid Stimulates Shoot and Bulb Formation of Garlic In Vitro. J Plant Growth Regul 12: 73–77.

[111] Zel J, Debeljak N, Ucman R, Ravnikar M (1997) The effect of jasmonic acid, sucrose and darkness on garlic (*Allium sativum* L.cv. ptujski jesenski) bulb formation *in vitro*. In Vitro Cell Dev. Biol – Plant 33: 231–235.

[112] Debeljak N, Regvar M, Dixon KW, Sivasithamparam K (2002) Induction of tuberisation *in vitro* with jasmonic acid and sucrose in an Australian terrestrial orchid, *Pterostylis sanguinea* . Plant Growth Regul 36: 253–260.

[113] Lee MW, Qi M, Yang Y (2001) A novel jasmonic acid-inducible rice myb gene associates with fungal infection and host cell death. Mol Plant Microbe Interact 14: 527–35. 11310740 10.1094/MPMI.2001.14.4.527

[114] Fernández-Calvo P, Chini A, Fernández-Barbero G, Chico JM, Gimenez-Ibanez S, Geerinck J, et al (2011) The Arabidopsis bHLH transcription factors MYC3 and MYC4 are targets of JAZ repressors and act additively with MYC2 in the activation of jasmonate responses. Plant Cell. 3: 701–15. 10.1105/tpc.110.080788 PMC3077776 21335373

[115] Meng XB; Zhao WS, Lin RM, Wang M, Peng YL (2005) Identification of a novel rice bZIP-type transcription factor gene, *OsbZIP1*, involved in response to infection of *Magnaporthe grisea* . Plant Mol Biol Rep 23: 301–302.

[116] Zhang HW, Lu XY, Huang DF, Huang RF (2004) The ethylene-, jasmonate-, abscisic acid- and NaCl-responsive tomato transcription factor JERF1 modulates expression of GCC box-containing genes and salt tolerance in tobacco. Planta 220: 262–270. 15300440 10.1007/s00425-004-1347-x

[117] Mueller MJ (1997) Enzymes involved in jasmonic acid biosynthesis. Physiol Plant 100: 653–663.

[118] Müller-Röber B, Sonnewald U, Willmitzer L (1992) Inhibition of the ADP-glucose pyrophosphorylase in transgenic potatoes leads to sugar-storing tubers and influences tuber formation and expression of tuber storage protein genes. EMBO 11: 1229–1238. 10.1002/j.1460-2075.1992.tb05167.x PMC556571 1373373

[119] Kuipers AGJ, Jacobsen E, Visser RGF (1994) Formation and Deposition of Amylose in the Potato Tuber Starch Granule Are Affected by the Reduction of Granule-Bound Starch Synthase Gene Expression. Plant Cell 6: 1 43–52. 10.1105/tpc.6.1.43 PMC160414 12244219

[120] Jackson SD, Heyer A, Dietze J, Prat S (1996) Phytochrome B mediates the photoperiodic control of tuber formation in potato. Plant J 9: 159–166.

[121] Carrera E, Bou J, García-Martínez JL, Prat S (2000) Changes in GA 20-oxidase gene expression strongly affect stem length, tuber induction and tuber yield of potato plants.Plant J. 22: 247–256. 10.1046/j.1365-313x.2000.00736.x 10849342

[122] Zeevaart JAD (2008) Leaf-produced floral signals. Cur Opin Plant Biol 11: 541–547. 10.1016/j.pbi.2008.06.009 18691931

[123] Baudry A, Ito S, Song YH, Strait AA (2010) Takatoshi Kiba,Sheen Lu, Rossana Henriques, José L. Pruneda-Paz, Nam-Hai Chua, Elaine M. Tobin, F-Box Proteins FKF1 and LKP2 Act in Concert with ZEITLUPE to Control Arabidopsis Clock Progression. Plant Cell 22: 606–622. 20354196 10.1105/tpc.109.072843 PMC2861467

[124] Krieger U, Lippman ZB, Zamir D (2010) The flowering gene single flower truss drives heterosis for yield in tomato. Nat Genet 42: 459–463. 20348958 10.1038/ng.550

[125] Imaizumi T, Kay SA (2006) Photoperiodic control of flowering: not only by coincidence. Trands in Plant Sci 11: 550–558. 10.1016/j.tplants.2006.09.004 17035069

[126] Banerjee AK, Chatterjee M, Yu Y, Suh SG, Miller WA, Hannapek DJ (2006) Dynamics of a mobile a RNA of potato involved in a long distance signaling pathway. Plant Cell 18: 3443–3457. 17189340 10.1105/tpc.106.042473 PMC1785412

[127] Kolomiets1 MV, Hannapel DJ, Chen H, Tymeson M, Gladon RJ (2001) Lipoxygenase is involved in the control of potato tuber development. Plant Cell 13: 613–626. 11251100 10.1105/tpc.13.3.613 PMC135504

[128] Müller-Röber B, Sonnewald U, Willmitzer L (1992) Inhibition of the ADP-glucose pyrophosphorylase in transgenic potatoes leads to sugar-storing tubers andinfluences tuber formation and expression of tuber storage protein genes. EMBO 11: 1229–12. 10.1002/j.1460-2075.1992.tb05167.x PMC556571 1373373

[129] Kuipers AGJ, Jacobsen E, Visser RGF (1994) Formation and Deposition of Amylose in the Potato Tuber Starch Granule Are Affected by the Reduction of Granule-Bound Starch Synthase Gene Expression. Plant Cell 6: 43–52. 12244219 10.1105/tpc.6.1.43 PMC160414

[130] Jackson SD (1996) Phytochrome B mediates the photoperiodic control of tuber formation in potato. Plant J 2: 159–166.

[131] Zeevaart JA (2008) Leaf-produced floral signals. Curr. Opin. Plant Biol. 11: 541–547. 10.1016/j.pbi.2008.06.009 18691931

