# Supplementary figures and images for: Genome-Wide Analysis of Differentially Expressed Genes Relevant to Rhizome Formation in Lotus Root (Nelumbo nucifera Gaertn)
Source: PLoS One. 2013 Jun 26;8(6):e67116. doi: 10.1371/journal.pone.0067116 (PMC3694149; doi:10.1371/journal.pone.0067116)

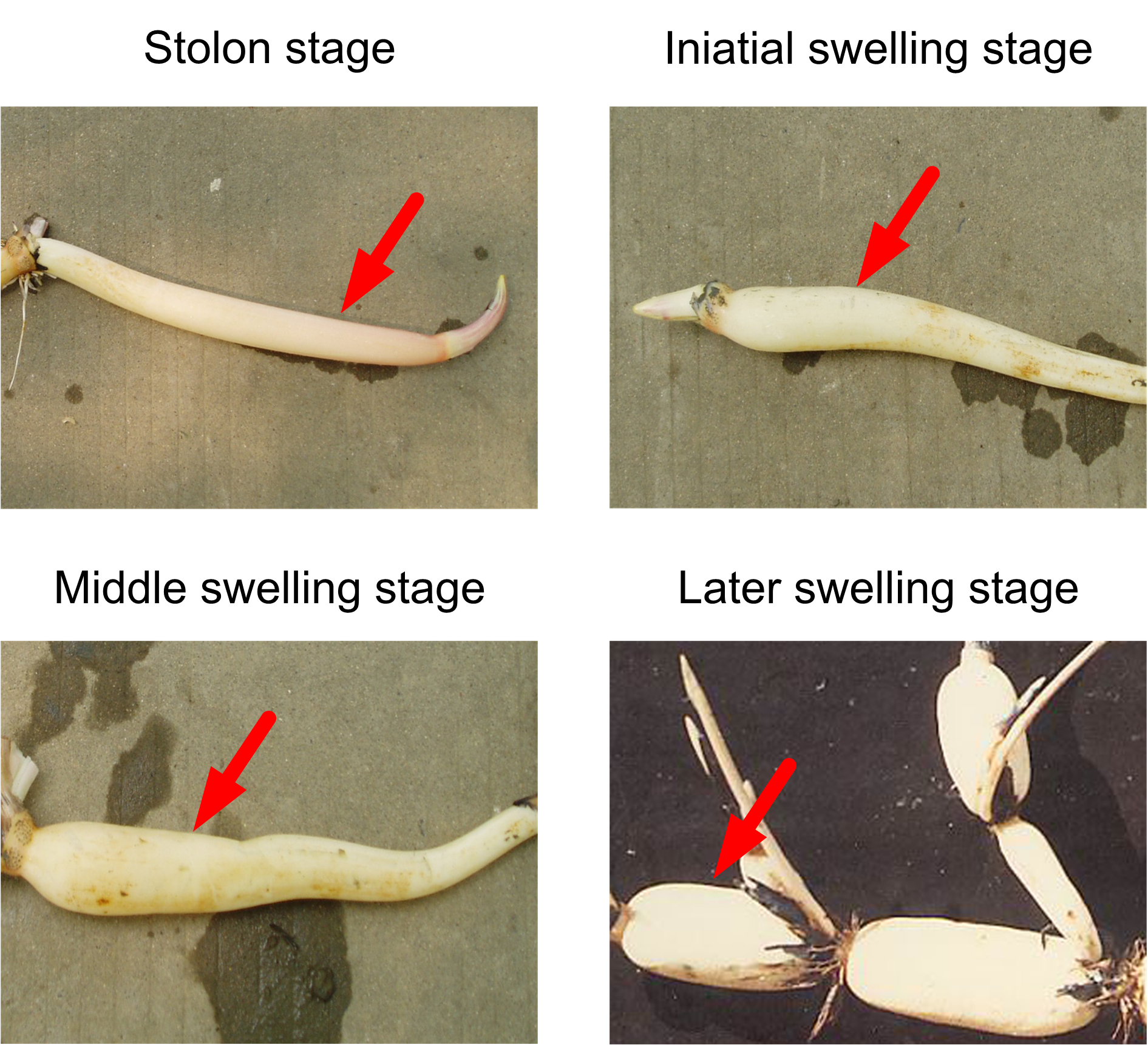

Supplement: Figure S1 — Developmental stages of lotus rhizome including stolon stage, initial stage, middle swelling stage and later swelling stage. (TIF) [file pone.0067116.s001.tif]

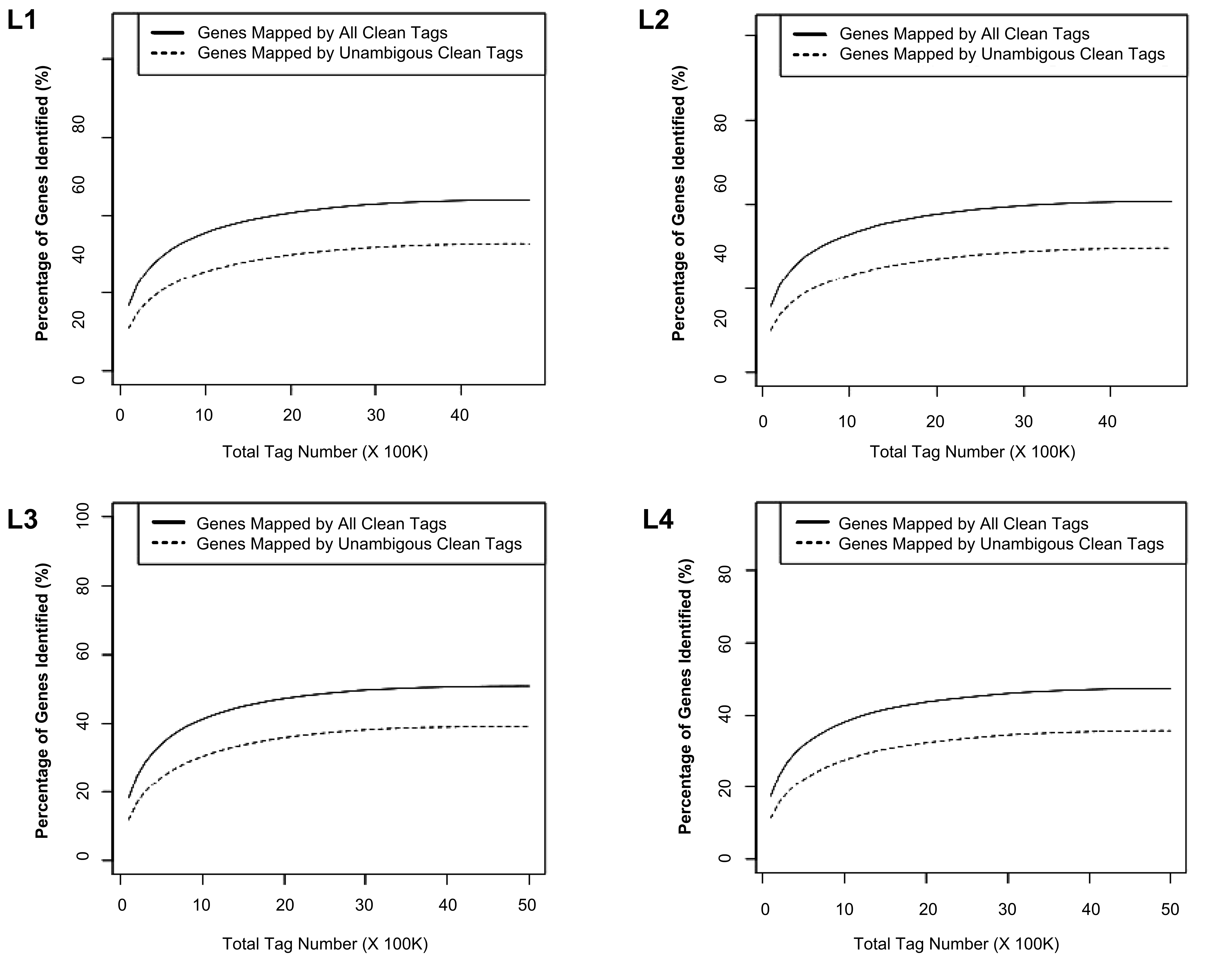

Supplement: Figure S2 — Sequencing saturation analysis of three libraries. L1: tag-sequencing for stolon stage; L2: tag-sequencing for initial swelling stage; L3: tag-sequencing for middle swelling stage; L4: tag-sequencing for later swelling stage. (TIF) [file pone.0067116.s002.tif]

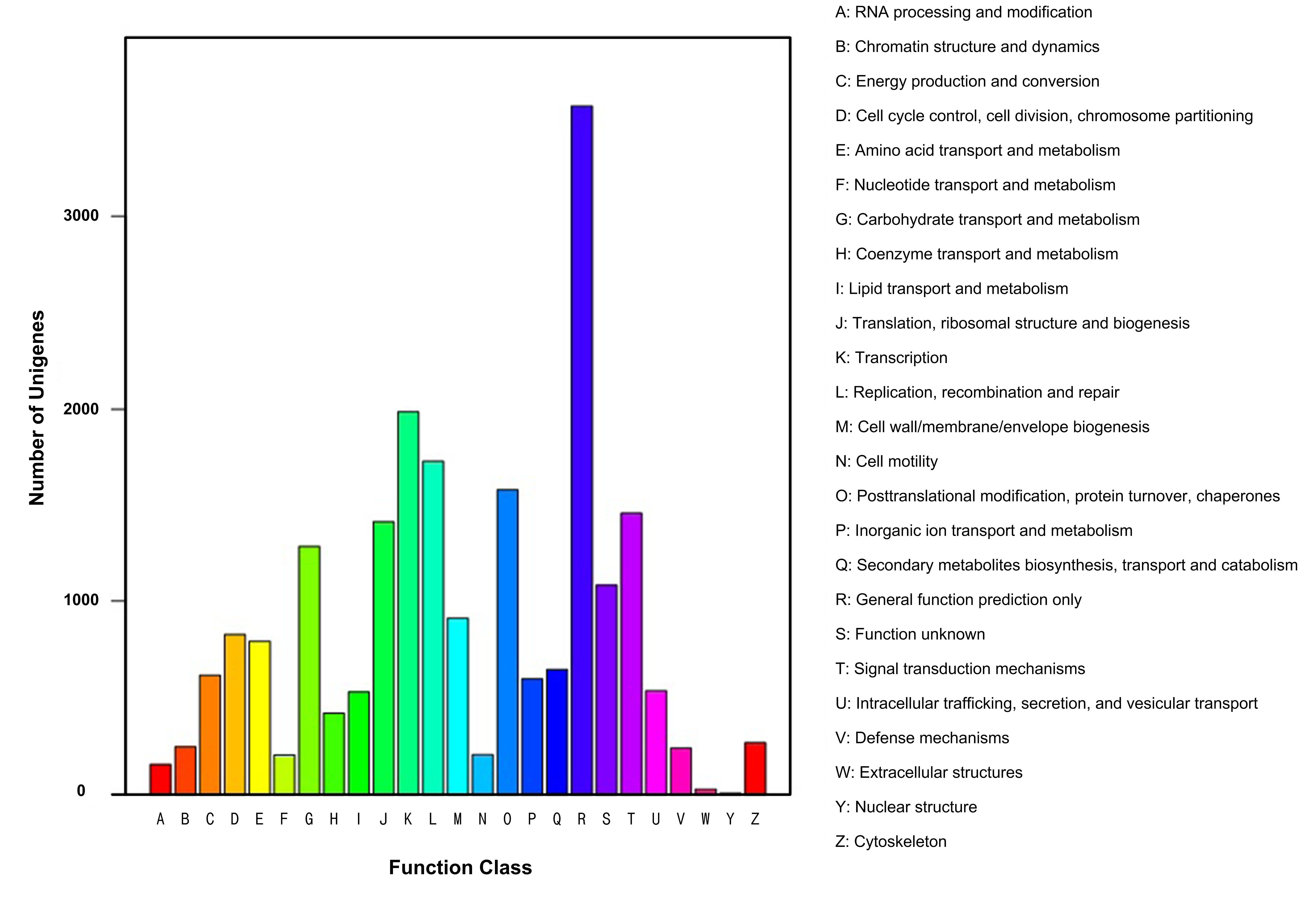

Supplement: Figure S3 — GO analysis of genes expressed during the rhizome formation. All the genes identified in L1/L2, L2/L3, and L3/L4 libraries were classified into 26 classifications according to gene functions. (TIF) [file pone.0067116.s003.tif]

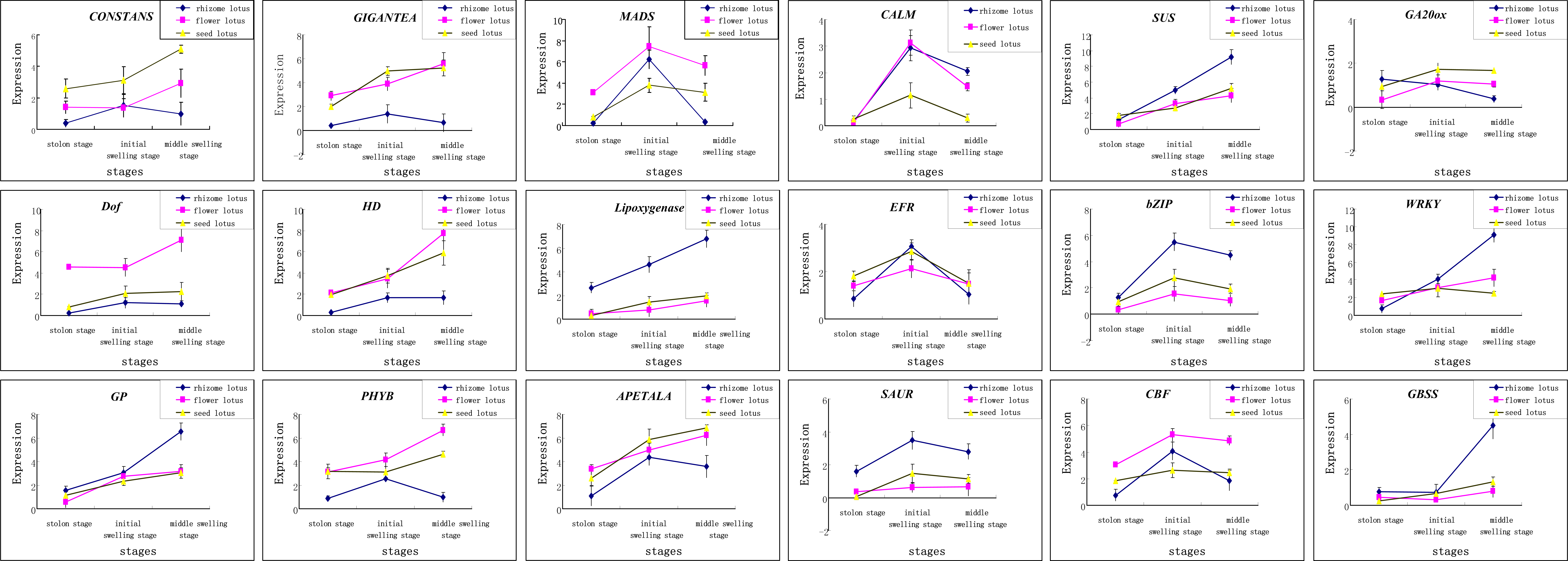

Supplement: Figure S4 — Expression profiles of 18 genes at stolon stage, initial swelling stage and middle swelling stage in rhizome lotus, flower lotus, and seed lotus. (TIF) [file pone.0067116.s004.tif]
